# Vibrational Disorder Effects on Temperature-Resolved X‑Ray Absorption Signatures of Metal Catalysts: From Single-Atoms to Clusters and Nanoparticles

**DOI:** 10.1021/acsnano.5c20042

**Published:** 2026-04-27

**Authors:** Wilson Henao, Iván López-Luque, Gonzalo Prieto, Giovanni Agostini

**Affiliations:** † 83167 Instituto de Tecnología Química (ITQ), Consejo Superior de Investigaciones Científicas-Universitat Politècnica de València, Spain. Av. Los Naranjos S/N, Valencia 46022, Spain; ‡ 18474 ALBA Synchrotron Light Source Carrer de la Llum2−26, Cerdanyola Del Vallès, Barcelona 08290, Spain; § Elettra-Sincrotrone, Basovizza, Trieste 34149, Italy

**Keywords:** EXAFS, vibrational disorder, single-atom catalysts, metal nanoclusters, operando spectroscopy, Debye−Waller factor

## Abstract

Revealing dynamic local-structure changes of (sub)­nanometric metal species under operating conditions is essential. In heterogeneous catalysis, this insight enables the rationalization of operation and optimization of catalyst efficiency and stability. Extended X-ray absorption fine structure (EXAFS) provides element-specific access to metal–metal coordination numbers, interatomic distances, and local disorder, which is pivotal when active motifs lack long-range order. Yet, accurate determination of structural parameters from EXAFS signatures is often complicated by the convolution of static heterogeneity and thermal vibration effects, encoded in the Debye–Waller factor: $\sigma^{2}=\sigma_{\mathrm{dynamic}}^{2}\left(T\right)+\sigma_{\mathrm{static}}^{2}$ . This coupling, especially at elevated temperatures typical of *in situ* and *operando* studies, obscures genuine structural changes. Here we present a temperature-resolved EXAFS study geared toward deconvoluting $\sigma_{\mathrm{dynamic}}^{2}$ ­(*T*) in three supported Ag catalysts spanning different $\sigma_{\mathrm{static}}^{2}$ levels and metal aggregation states: Al_2_O_3_-supported Ag nanocrystals, few-atom Ag clusters confined to a zeotype host, and single-atom Ag dispersed on WO_*x*_/Al_2_O_3_. Over 298–723 K, representative of catalyst activation and deployment conditions, we observe a nuclearity-dependent vibrational stiffness: Ag–Ag bonds in nanoparticles show strong thermal disorder, whereas Ag–O bonds in single-atoms and confined clusters remain comparatively rigid, limiting dynamic fluxionality. While a classical formalism, such as the correlated Einstein model, adequately captures nanocrystal dynamics, it fails for few- and single-atom motifs. Therefore, a direct parametrization of σ^2^(*T*) is proposed, better capturing vibrational disorder in low-nuclearity metal catalysts. The results provide guidance for decoupling thermal and static contributions in temperature-resolved EXAFS studies, enabling a more reliable structural analysis of (sub)­nanometric metal species under *operando* conditions.

## Graphical abstract

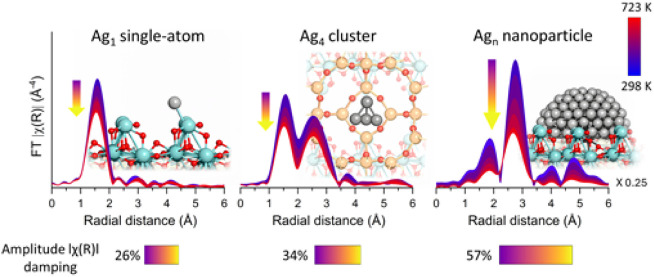

## Introduction

Functional metal species dispersed on, or stabilized within, support materials are ubiquitous across technologies, from optics^1,2^ and sensing^3,4^ to energy conversion^5,6^ and catalysis.^7–10^ In catalysis, supported metal catalysts are central to a wide variety of processes, from environmental protection to the production of fuels and synthetic energy carriers, or the synthesis of commodity chemicals from both fossil and renewable feedstocks. Particularly in the last decades, growing attention has been placed on designing and optimizing low-atomicity metal catalysts, i.e., those based on subnanometer clusters and, in the limit of atomic dispersion, mononuclear complexes or “single-atom” sites.^11–17^ The rational development of such catalysts with precisely controlled nuclearity requires parallel advances in structural characterization, particularly under conditions that replicate real operating environments.

A suite of complementary spectroscopies has been developed to elucidate the structure of subnanometer metal assemblies that lack long-range order.^18–20^ Aberration-corrected electron microscopy enables real-space imaging of individual atoms in supported metal catalysts with atomic resolution.^21^ However, its application can be limited by factors such as e-beam-induced damage, the requirement to operate under ultrahigh vacuum conditions (markedly departing from catalyst activation and operation conditions), and the limited statistical sampling due to the small probed sample volume. Infrared spectroscopy coupled to probe molecules samples larger sample volumes and reports on surface adsorbate-metal interactions under reaction conditions,^22^ yet it provides indirect, ligand-specific information and cannot unambiguously distinguish metal–metal from metal–support bonding. Solid-state nuclear magnetic resonance (NMR)^23^ and Mössbauer^24^ spectroscopies yield insights into electronic structure and site symmetry, but only for NMR- or Mössbauer-active nuclei. Isotopic enrichment is often required, and true *operando* measurements are often challenging.

Among these methods, extended X-ray absorption fine-structure (EXAFS) spectroscopy stands out for its ability to provide direct, element-specific quantification of the local atomic environment around metal centers.^25–27^ By combining atomic-scale and high time resolutions with bulk analysis depths, EXAFS affords statistically robust characterization of all classes of metal catalysts, including those with low-atomicity metal species lacking long-range atomic ordering. Moreover, owing to its compatibility with a diversity of sample *operando* environments (composition, temperature, pressure), it uniquely enables real-time monitoring of dynamic structural changes under realistic process conditions.^28–33^

The EXAFS signal, denoted as χ­(*k*), arises from the interference between the outgoing photoelectron wave emitted by an absorber atom and the waves scattered by its neighboring atoms in the j-th coordination shell. Quantitative structural information, such as interatomic distances (*R*), coordination numbers (*N*), and local disorder (usually expressed as the Debye–Waller factor, σ^2^) can be extracted by fitting experimental EXAFS spectra to theoretical models based on a sum of scattering contributions from simulated photoelectron scattering paths.^34–36^ In practice, however, the reliable determination of these parameters is often hindered by strong intrinsic correlations, most notably between *N* and σ^2^. Both parameters impose simultaneous yet opposing effects on the amplitudes of the EXAFS oscillations: *N* increases the amplitude linearly, whereas σ^2^ reduces it exponentially as a function of *k*. As a result, a reduction in EXAFS amplitude may arise either from a decrease in coordination number or from an increase in local disorder, making these effects difficult to disentangle, particularly when only spectra with a short *k*-range are available. This ambiguity contributes to a greater uncertainty of the fit, rendering changes in *N* statistically unreliable. This stands as a critical limitation for resolving subtle structural dynamics in catalysts with low metal nuclearity.^37^

The challenge is aggravated under temperature-resolved, *in situ*, or *operando* measurements, where thermal excitation intensifies atomic vibrations, causing dynamic fluctuations in atomic positions and, consequently, pronounced temperature-dependent variations in the extracted structural parameters.^38,39^ In low-atomicity catalysts, additional complexity arises from the broad distribution of atomic configurations around ultrasmall metal centers, which leads to significant structural disorder deviating from ideal periodic coordination environments.^40^ Both thermal (dynamic) and structural (static) disorder effects are captured by the Debye–Waller factor, $\sigma^{2}=\sigma_{\mathrm{dynamic}}^{2}\left(T\right)+\sigma_{\mathrm{static}}^{2}$ .^41,42^ Disentangling these contributions is crucial for resolving genuine structural transformations—such as aggregation, redispersion, or adsorbate-induced metal restructuring—that might otherwise be masked by concurrent thermally driven disorder.

To capture thermal effects, EXAFS analyses routinely incorporate models of atomic vibrations.^43,44^ The Debye model treats the entire solid as an isotropic elastic continuum, where all atoms vibrate collectively with a distribution of frequencies up to a maximum cutoff (the Debye frequency).^45^ It effectively captures the temperature dependence of atomic displacements in isotropic crystalline systems. However, it assumes uncorrelated motion between atom pairs, which limits its accuracy for short-range order systems. The correlated Einstein model improves on this by modeling atom pairs as coupled harmonic oscillators vibrating with a single effective frequency, *ω_E_*.^46^ It considers the relative motion between absorber and backscatterer atoms, thereby accounting for the partial correlation in their displacements. This makes the correlated Einstein model better suited for describing dynamic disorder in systems where individual bond vibrations are more localized and directional.^47^

While these models are useful for many crystalline or moderately disordered systems, they often fail for highly dispersed catalysts where anharmonic and anisotropic vibrations dominate. To address these circumstances, cumulant expansion techniques have been introduced by expressing the EXAFS function in terms of statistical cumulants *C_i_* to describe deviations from a purely Gaussian bond-length distribution: *C*_1_ (average bond length), *C*_2_ (mean-square relative displacement), *C*_3_ (asymmetry or skewness), and *C*_4_ (sharpness or kurtosis).^48,49^ However, the incorporation of higher-order cumulants increases the number of free parameters in the model, which can induce new correlations and reduce the robustness of the fit.

These conceptual frameworks have attempted to underpin a warning that an explicit, physically motivated treatment of the Debye–Waller factor is essential to quantitatively correct structural parameters extracted from temperature-dependent EXAFS measurements, which are otherwise biased by thermal disorder. Although this consideration has been extensively addressed in investigations of crystalline solids, only a limited number of studies have systematically incorporated temperature-dependent σ^2^(*T*) models in the analysis of EXAFS spectra of nanoscale and highly dispersed systems.

In carbon-supported Pt nanoparticles, Frenkel et al.^41^ demonstrated that applying a correlated Einstein model for σ^2^(*T*) and including anharmonic effects via the *C*_3_ cumulant eliminates an apparent, nonphysical contraction of the nearest-neighbor Pt–Pt bond at elevated temperatures (473–673 K). Proper σ^2^(*T*) treatment revealed a size-dependent $\sigma_{\mathrm{static}}^{2}$ disorder term, increasing with decreasing particle size from 0.0005 Å^2^ in bulk Pt to 0.0017 Å^2^ in 2 nm nanoparticles, attributed primarily to surface bond relaxation rather than uniform lattice strain. Extending this analysis to *operando*-relevant conditions under an H_2_ atmosphere up to 673 K, Bus et al.^48^ showed that omission of higher-order cumulants in T-EXAFS spectra of supported ∼1 nm Pt clusters induced severe artifacts in *R* and Δ*E*_0_ (up to 0.08 Å and 10 eV). They further confirmed that non-Gaussian disorder biases both σ^2^ and coordination number, leading to errors of up to ∼20–40% in *N* and Δσ^2^ ≈ 0.006 Å^2^. Enforcing a linear σ^2^(*T*) and including *C*_3_ and *C*_4_ cumulants, the Pt–Pt distance stabilized to near physically reasonable values (2.72–2.73 Å), while preserving nearly constant coordination numbers (*N* = 5–7). Similar studies on Au nanoparticles^50^ and on carbon-supported Rh clusters^38^ further corroborate that non-Gaussian disorder is intrinsic to nanoscale metals, leading to suppressed or even negative thermal expansion and systematic underestimation of *N* and σ^2^ unless *C*_3_ and/or *C*_4_ are included. More recently, Øien et al. reported *operando* TPR-EXAFS measurements on isolated Pt sites in UiO-67,^37^ showing that standard single-spectrum analysis leads to nonphysical Debye–Waller factors, including negative σ^2^ values, and spurious increases in Pt–N coordination at temperatures above 600 K. By refining the full temperature series using a parametric σ^2^(*T*) approach based on the Einstein model, physically meaningful σ^2^ values were recovered, enabling reliable quantitative tracking of ligand removal and Pt framework anchoring without invoking higher-order cumulants. A similar strategy was later applied by Kang et al.^51^ to isolated atomic Cu sites anchored on CeO_2_ during CO oxidation.

In this study, we examine the ability of different models to capture the temperature dependence of σ^2^(*T*) across supported catalysts ranging from nanoparticles to clusters and single atoms, providing insight into metal-coordination-dependent vibrational stiffness. We show that changes in nuclearity and coordination environment across this series of metal assemblies introduce different levels of $\sigma_{\mathrm{static}}^{2}$ , *e.g., via* under-coordination or broadened bond-length distributions. To isolate the temperature-dependent $\sigma_{\mathrm{dynamic}}^{2}$ ­(*T*) component associated with thermally activated vibrations, we verified that the catalysts preserved their structural integrity during thermal treatment. The minimal changes observed in metal–metal coordination numbers and interatomic distances support the working assumption that $\sigma_{\mathrm{static}}^{2}$ remains effectively constant over the temperature window examined for each catalyst. Under these conditions, variations in σ^2^ can primarily be attributed to $\sigma_{\mathrm{dynamic}}^{2}$ ­(*T*). Applying three modeling strategies, namely unconstrained σ^2^(*T*) fitting, the correlated Einstein model σ^2^(*T*; *θ_E_*), and an empirical linear parametrization σ^2^(*T*; *α*, *β*), allowed the evaluation of the relative contributions of $\sigma_{\mathrm{static}}^{2}$ and $\sigma_{\mathrm{dynamic}}^{2}$ ­(*T*) to the total Debye–Waller factor σ^2^ for each system at a given temperature.

To experimentally access these nuclearity- and coordination-dependent effects, synthesis methods were carefully controlled to obtain catalyst materials featuring Ag nanocrystals supported on Al_2_O_3_, subnanometer few-atom Ag clusters confined within a zeotype host, and atomically dispersed Ag sites on WO_*x*_/Al_2_O_3_, offering a systematic platform to probe the relationship between local structural characteristics and thermal disorder. Supported silver catalysts were selected as a technologically and mechanistically relevant case study due to their broad industrial relevance and emerging opportunities in low-atomicity catalysis.^52^ Nanoparticulate Ag catalysts are relevant for industrial processes such as ethylene epoxidation to ethylene oxide,^53–55^ selective hydrogenation reactions,^56,57^ as well as selective NH_3_ oxidation.^58^ In parallel, low-atomicity Ag catalysts, including nanoclusters and single-atom Ag species, are gaining increasing attention for processes such as alkyne carboxylation with CO_2_,^59,60^ direct propylene epoxidation,^61^ and (photo)­oxidation processes for environmental protection.^62–64^

## Results and Discussion

### Catalysts’ Structural Characterization

Our study relies on a suite of Ag-based model-supported catalysts synthesized to display controlled nuclearity, spanning large nanoparticles, subnanometer few-atom clusters, and atomically dispersed single atoms. A schematic overview of these architectures is provided in Figure 1a,d,g. The metal aggregation state of Ag in the catalysts was systematically assessed through a multiscale analysis combining aberration-corrected high-angle annular dark-field (HAADF) and integrated differential phase contrast (iDPC) scanning transmission electron microscopy (STEM), X-ray diffraction (XRD), and X-ray absorption spectroscopy (XAS).

**Figure 1:**
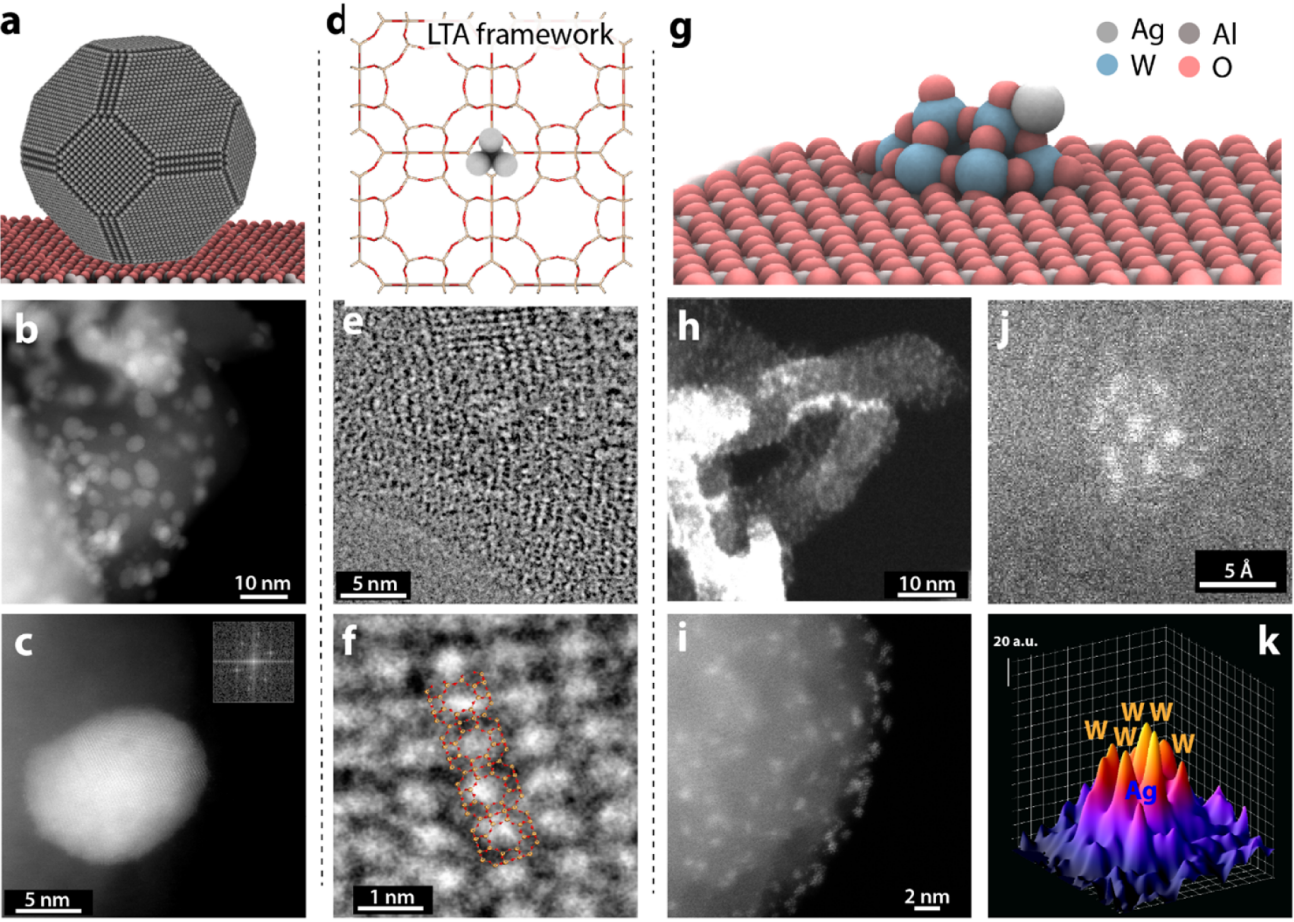
Electron microscopy characterization of Ag-based supported catalysts with different Ag atomicity. Panels a, d, and g show schematic models for: a) Ag_np_/Al_2_O_3_ catalyst containing crystalline Ag nanoparticles supported on α-Al_2_O_3_; d) Ag_clus_/LTA catalyst with few-atom Ag nanoclusters confined within the framework of LTA zeolite; and g) Ag_1_–WO*_x_*/Al_2_O_3_ catalyst with Ag atomically dispersed and stabilized by polytungstate WO*_x_* clusters on a γ-Al_2_O_3_ carrier. b,c) HAADF-STEM micrographs for Ag_np_/Al_2_O_3_ catalyst. The inset to panel c shows the fast Fourier Transform (FFT) recorded at the high-contrast Ag nanoparticle, showing its crystalline character. e,f) Integrated Differential Phase Contrast (iDPC) micrographs for Ag_clus_/LTA. In panel f, the framework of the LTA zeolite is superimposed to facilitate interpretation. h,i) HAADF-STEM micrographs for Ag_1_–WO*_x_*/Al_2_O_3_ showing high Z-contrast WO*_x_* clusters decorating the outer surface of ellipsoidally shaped γ-Al_2_O_3_ crystallites. Panels j and k show a detailed HAADF-STEM micrograph for a supported Ag_1_–WO*_x_* nanocluster and the corresponding Z-contrast 3D map with the proposed identification of individual atoms.

Aberration-corrected HAADF-STEM and iDPC-STEM analyses provided insights into the spatial distribution and dispersion of Ag species. HAADF-STEM imaging of Ag_np_/Al_2_O_3_ catalyst revealed crystalline Ag nanoparticles ranging from 5 to 25 nm in size, deposited on the outer surface of α-Al_2_O_3_ grains (Figure 1b,c), exemplifying a conventional supported metal catalyst where the active phase exists as relatively large metallic crystallites. In contrast, no large metal aggregates were detected in the Ag_clus_/LTA sample. Moreover, this catalyst exhibited significant instability under electron-beam irradiation. Even short exposure led to rapid amorphization of the zeolitic framework and the sintering of initially undetectable Ag atoms into small Ag aggregates (<2 nm), which remained confined within the aluminosilicate support’s porosity (Figure S1). Such instability, even at low electron doses, is known in low Si/Al ratio zeolites.^65^ LTA is notably one of the most electron beam-sensitive zeolites, attributed to its high lattice aluminum content (Si/Al ≈ 1) and low framework density (12.9 (Si + Al) atoms per 1000 Å^3^).^66^ Consequently, reliable imaging of the pristine Ag species in Ag_clus_/LTA was not feasible. Under iDPC-STEM conditions, the sample also underwent rapid amorphization; however, degradation was slower, allowing the imaging of crystalline domains that retained the LTA zeolite framework structure (Figures 1e,f). A higher contrast in regions corresponding to the sodalite cages of the LTA structure suggested the localization of Ag in these crystallographic positions (Figure 1f). However, further elucidation of the Ag aggregation state was not possible through direct imaging of this material. Finally, the Ag_1_–WO_*x*_/Al_2_O_3_ catalyst was examined by HAADF-STEM. As shown in Figure 1h,i, small WO_*x*_ clusters (<3 nm) decorated the surface of ovoid-shaped γ-Al_2_O_3_ crystallites. Atomic-resolution imaging combined with a local Z-contrast mapping analysis (Figures 1j,k) confirmed a polytungstate-like structure. Alongside high-Z W atoms (Z_W_ = 74), isolated, smaller, and lower Z-contrast Ag atoms (Z_Ag_ = 47) were also detected in association with the WO_*x*_ polytungstate clusters. The results indicate that in this catalyst, single Ag atoms are stabilized on WO_*x*_ nanoclusters, lacking direct Ag–Ag coordination.

To complement the localized STEM observations with bulk-averaged structural information, XRD analysis was conducted (Figure S2). Ag_np_/Al_2_O_3_ exhibited characteristic diffraction peaks corresponding to the *Fm3m* phase of metallic silver. In contrast, no reflections attributable to metallic or oxidic Ag phases were detected in Ag_clus_/LTA and Ag_1_–WO_*x*_/Al_2_O_3_, consistent with the presence of Ag in highly dispersed forms lacking long-range atomic order.

The local coordination environment and metal nuclearity of the as-synthesized catalysts were further assessed by EXAFS analysis at the Ag K-edge using simulated scattering paths for Ag–Ag and Ag–O interactions, refined to capture the distinct local structure of each system (Figure 2). Detailed quantitative fitting results are provided in Figure S3 and Table S1. In Ag_np_/Al_2_O_3_, the FT-|χ­(R)| spectrum displayed a prominent Ag–Ag coordination peak at ∼2.8 Å, along with additional scattering features at higher radial distances, characteristic of a well-ordered crystalline lattice (Figure 2b). First-shell fitting analysis confirmed a full Ag–Ag coordination (*N* = 12 ± 0.1) with a bond length of 2.868 ± 0.008 Å and a moderate structural disorder (σ^2^ = 0.013 ± 0.002 Å^2^), consistent with Ag bulk-like *fcc* nanocrystals.^67^

**Figure 2:**
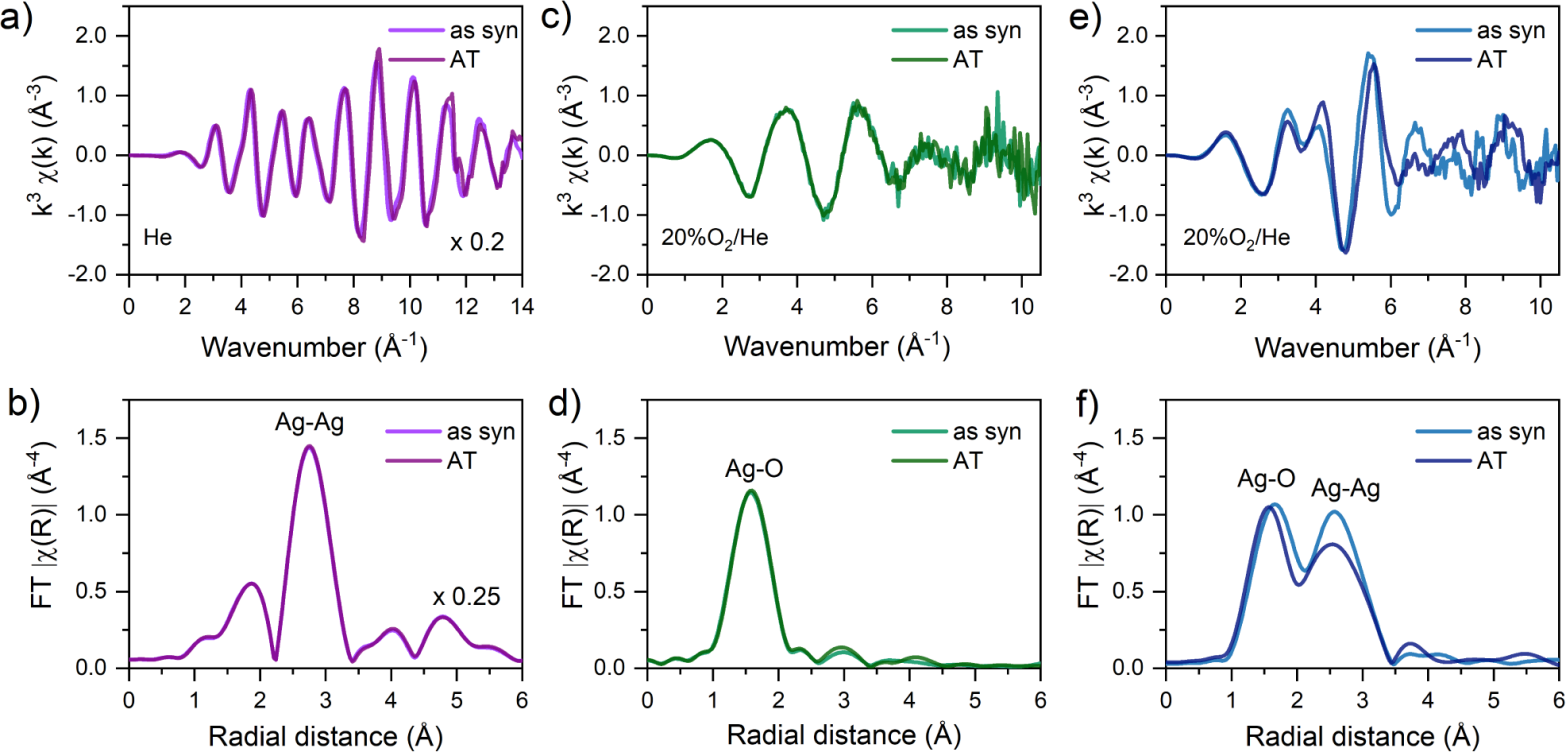
Ag K-edge EXAFS signals of the as-synthesized materials (as-syn) and their structural stability after the thermal treatment (AT). *k* ^3^-χ­(*k*) EXAFS spectra and their corresponding Fourier transforms FT-|χ­(R)| (uncorrected for phase shift) for the model catalysts: a,b) silver nanoparticles (Ag_np_/Al_2_O_3_); c,d) silver single atoms (Ag_1_–WO*_x_*/Al_2_O_3_); and e,f) silver clusters (Ag_clus_/LTA). The spectra were collected at 298 K on the as-synthesized materials before (as syn) and after (AT) thermal treatment. The Fourier transforms were extracted using a Hanning apodization window over the *k*-range of 1.0–8.0 Å^–1^.

On the other hand, Ag_1_–WO_*x*_/Al_2_O_3_ exhibited a single peak at ∼1.9 Å, corresponding to Ag–O interactions with the polytungstate WO_*x*_ matrix (Figure 2d). The low Ag–O coordination number (*N* = 3.2 ± 0.7) indicated undercoordinated Ag sites, while the absence of further Ag–O or Ag–Ag scattering contributions at higher radial distances confirmed their atomically dispersed nature. In addition, the large disorder parameter (σ^2^ = 0.023 ± 0.007 Å^2^) and the elongated bond length (Δ*R* = 0.15 ± 0.03 Å) revealed a highly heterogeneous coordination environment around Ag atoms, characteristic of isolated metal atoms dispersed on oxide surfaces.^68,69^

Ag_clus_/LTA showed a more complex FT-|χ­(R)| profile with two main scattering peaks around ∼1.9 and ∼2.8 Å, attributed to Ag–O and Ag–Ag coordination shells, respectively (Figure 2f). The Ag–O contribution (*N* = 3.0 ± 0.8) indicated coordination to water ligands (*R*_Ag–OH_2__= 2.29 ± 0.02 Å) from the hydrated zeolite framework, while the Ag–Ag scattering (*N* = 3.1 ± 1.3) revealed the presence of tetrahedral-like Ag_4_ assemblies (*R*_Ag–Ag_ = 2.80 ± 0.03 Å).^70^ As suggested by HAADF-STEM imaging and proposed in earlier studies,^70^ these low-nuclearity Ag_4_ clusters are preferentially located in the sodalite cavities of the LTA zeolite framework. This confinement is consistent with the low-intensity FT-|χ­(R)| peak around ∼3.7 Å, which is assigned to noncovalent coordination of Ag atoms with Si/Al atoms in the zeolite framework.^71,72^ Notably, the Ag–Ag bond in the Ag_4_ clusters was contracted by −0.09 ± 0.03 Å relative to bulk metallic silver, accompanied by a moderate level of static disorder (σ^2^ = 0.017 ± 0.009 Å^2^). Spatial confinement of metal clusters often leads to such structural deviations from the bulk phase due to reduced nuclearity and strong host–guest interactions.^73,74^

The structural stability of the catalysts under thermally relevant conditions was evaluated by heating the materials to 723 K. These thermal treatments were performed under gas atmospheres selected to prevent changes in the metal aggregation state. In the case of Ag_clus_/LTA and Ag_1_–WO_*x*_/Al_2_O_3_catalysts, a flow of 20% O_2_/He was applied to prevent reductive aggregation phenomena from the low-atomicity Ag species. Conversely, for Ag_np_/Al_2_O_3_ the flowing gas was inert helium to preclude oxidative crystallite disruption and metal redispersion.^75–77^ Figure 2 compares the EXAFS spectra collected at room temperature (298 K) on the as-synthesized materials before and after thermal treatment.

The FT-|χ­(R)| spectra of Ag_np_/Al_2_O_3_ and Ag_1_–WO_*x*_/Al_2_O_3_ remained unchanged following the thermal treatment (Figure 2b,d), highlighting the preservation of the original metal aggregation state. In the case of Ag_1_–WO_*x*_/Al_2_O_3_, strong Ag–O interactions were key to suppressing both migration and agglomeration of Ag_1_ atoms. For Ag_np_/Al_2_O_3_, the inert helium atmosphere helped preserve the integrity of the extended metallic crystallites, with no modifications detected in the EXAFS features after the temperature-resolved experiment. In both catalysts, the coordination numbers N_Ag–Ag_ and N_Ag–O_ of the Ag species and the corresponding radial distances to neighboring atoms were maintained, demonstrating the preservation of their local structure with no alterations in the local disorder (σ^2^) after heating (Table S1).

In contrast, variations in the FT-|χ­(R)| peak intensities of Ag_clus_/LTA indicated a structural rearrangement of the confined Ag_4_ clusters (Figure 2f), likely driven by the mobility of metal species during zeolite dehydration occurring up to ∼500 K (Figure S4).^78^ As water is removed from the framework, the reduced steric and electrostatic constraints facilitate Ag–Ag coalescence, as evidenced by the increase in *N*_Ag–Ag_ from 3.1 ± 1.3 to 4.7 ± 1.7, indicating the growth of pristine Ag_4_ clusters into a more compact, octahedral-like Ag_6_ structure upon heating. The increase in the coordination number was accompanied by a rise in σ^2^_Ag–Ag_ from 0.017 ± 0.009 Å^2^ to 0.027 ± 0.009 Å^2^ reflecting greater local disorder (Table S1). Once zeolite dehydration is complete, the Ag_6_ clusters maintained their structural integrity, with a constant *N*_Ag–Ag_ = 4.9 ± 0.7 confirmed by an internal corefinement fitting from 473 to 723 K (Table S5). These results are consistent with prior reports that dehydrating LTA zeolites initially containing Ag_4_ clusters yields Ag_6_ assemblies, and that the resulting increase in metal nuclearity directly modulates their photoluminescence response.^70,79,80^

To demonstrate the functional relevance of the structurally characterized species, model catalytic reactions were performed. Specifically, Ag_np_/Al_2_O_3_ was evaluated for ethylene epoxidation, a benchmark industrial reaction for nanoparticulate Ag catalysts, while Ag_1_–WO_*x*_/Al_2_O_3_ was tested in the carboxylation of phenylacetylene with CO_2_, a reaction known to benefit from isolated, electronically deficient Ag sites. In both cases, the observed catalytic activity performance (Table S2) agreed well with that expected for comparatively large Ag nanocrystals and Ag_1_ single-atom catalysts, respectively, confirming that the metal species probed by EXAFS are catalytically competent and structurally relevant under reaction environments.

### Temperature-Resolved EXAFS: Thermal Disorder in Supported Metal Catalysts

To probe the interplay between thermal excitation and vibrational disorder, we conducted temperature-resolved EXAFS using a stepwise heating protocol from 298 to 723 K (Figure 3). In all three cases, the *k* ^3^-weighted χ­(*k*) oscillations diminished progressively with temperature (Figure 3a,c,e), directly reflecting the damping of photoelectron scattering by atomic vibrations.

**Figure 3:**
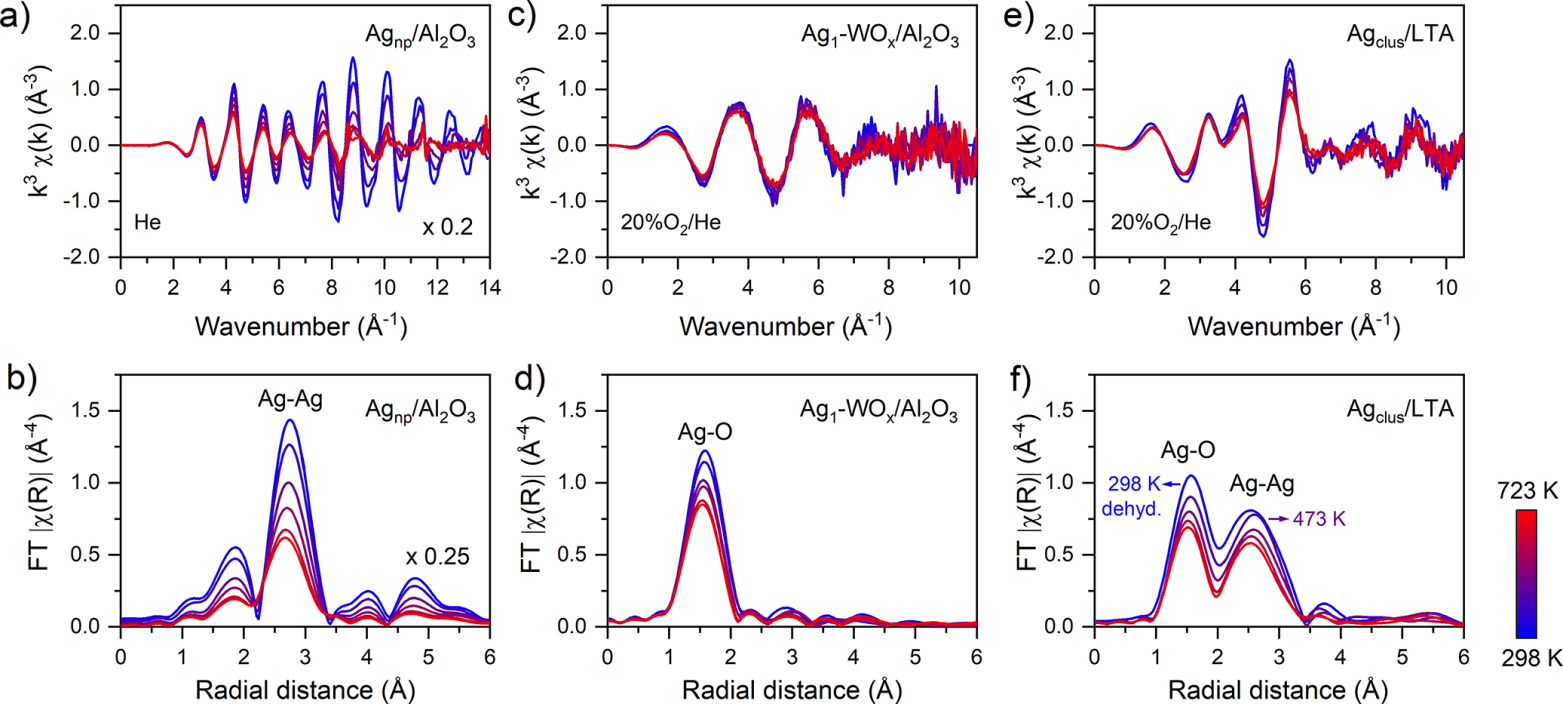
Temperature effects on the EXAFS signature. *In situ* temperature-resolved *k* ^3^-weighted χ­(*k*) functions in reciprocal *k*-space (top) and their corresponding Fourier transforms FT-|χ­(R)| (uncorrected for phase shift, bottom) for the model catalysts: a,b) silver nanoparticles (Ag_np_/Al_2_O_3_); c,d) silver single atoms (Ag_1_–WO*_x_*/Al_2_O_3_); and e,f) silver few-atom clusters (Ag_clus_/LTA). The Fourier transforms were extracted using a Hanning apodization window over the *k*-range of 1.0–8.0 Å^–1^.

Given the structural stability of the materials during thermal treatment, the influence of sintering and thermal expansion can be neglected, indicating that the observed damping primarily originates from thermal effects.^41,42^ As the temperature rises, atoms vibrate with greater amplitude around their equilibrium positions, leading to a larger mean-square relative displacement between neighboring atoms. This harmonic motion attenuates the EXAFS signal through the Debye–Waller factor σ^2^ in the $e^{-2k^{2}\sigma_{j}^{2}}$ term, particularly at high *k*-values where the phase shift (Δϕ = 2*k*Δ*r*) becomes highly sensitive to positional fluctuations. Anharmonic effects and phonon excitations further contribute to dynamic fluctuations in bond lengths, increasing inelastic scattering and reducing photoelectron wave coherence.^65^

In the Fourier-transformed EXAFS spectra, these thermal effects were evidenced by peak broadening and reduction in the |χ­(*R*)| amplitude (Figures 3b,d,f). Apparent peak shifts toward shorter distances were also observed, reflecting temperature-dependent phase alterations (Δϕ).^81^

The extent of EXAFS signal attenuation was strongly influenced by the metal nuclearity and the local coordination environment. In Ag_np_/Al_2_O_3_, the scattering amplitude of the Ag–Ag peak at ∼2.8 Å decreased by 57% at 723 K relative to the spectrum at 298 K (Figure 3b). This pronounced damping indicated the increased vibrational freedom of Ag atoms within the nanoparticle lattice, a consequence of delocalized metallic bonding that yields weak restoring forces and increased thermal disorder. For comparison, EXAFS measurements on an Ag metal foil (25 μm thick) reported only a 37% decrease in the FT-|χ­(R)| peak amplitude over the range 294–820 K, underscoring the superior lattice cohesion of the extended crystalline bulk metal relative to its nanoscale counterpart.^82,83^

Ag_1_–WO_*x*_/Al_2_O_3_ exhibited higher thermal resilience, showing only a 26% reduction in the amplitude of the Ag–O scattering peak (Figure 3d). Despite the undercoordination of Ag atoms in this material, the rigid Ag–O bonding environment effectively restricted thermal motion, thereby limiting atomic displacements and mitigating EXAFS signal attenuation. Ag_clus_/LTA showed an intermediate response, where the collective Ag–Ag vibrational modes of the subnanometric Ag_6_ clusters partially coupled to the lattice dynamics of the LTA framework, limiting atomic flexibility. As a result, moderate FT-|χ­(*R*)| damping was observed, with amplitude reductions of 34% and 28% for Ag–O and Ag–Ag shells, respectively (Figure 3f).

To quantitatively assess the temperature-dependent evolution of thermal disorder in the Ag K-edge EXAFS spectra, the Debye–Waller factors (σ^2^) were extracted from FEFF-based analysis using three different modeling strategies: (i) unconstrained σ^2^(*T*) fitting, (ii) correlated Einstein model σ^2^(*T*; *θ_E_*), and (iii) linear parametrization σ^2^(*T*; *α*, *β*). The results are summarized in Figure 4.

**Figure 4:**
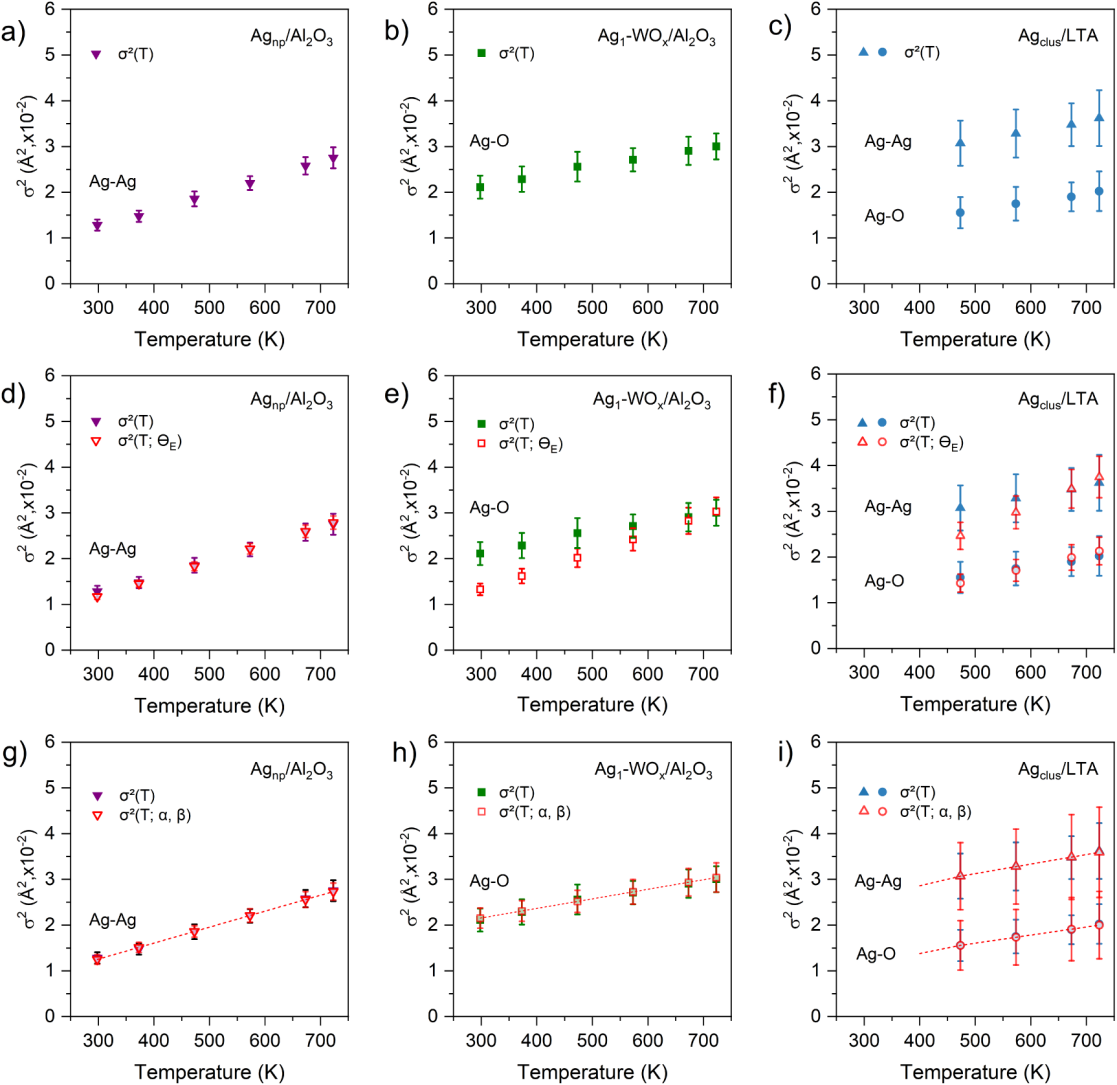
Description of the temperature-dependent component in the Debye–Waller factor (σ^2^) for Ag catalysts of different metal aggregation levels: a–c) unconstrained σ^2^(*T*) fitting; d–f) correlated Einstein model σ^2^(*T*; *θ_E_*); and g–i) linear parametrization σ^2^(*T*; *α*, *β*).

Under the unconstrained σ^2^(*T*) model, σ^2^ increased monotonically with the temperature for all materials, consistent with the expected rise in vibrational disorder (Figure 4a–c). Yet, the absolute values and temperature dependence of σ^2^ varied markedly across systems. When comparing the baseline, Ag_np_/Al_2_O_3_ exhibited the lowest disorder, from 0.013 Å^2^ at 298 K to 0.028 Å^2^ at 723 K, reflecting the coherent lattice of the crystalline nanoparticles. In contrast, σ^2^ values for Ag_1_–WO_*x*_/Al_2_O_3_ were consistently higher (0.021 Å^2^ to 0.030 Å^2^) likely due to greater local heterogeneity in Ag–O coordination. Ag_clus_/LTA showed the highest disorder in Ag–Ag paths (σ^2^ = 0.031–0.036 Å^2^), indicating the flexibility of the metal bonds within the Ag_6_ clusters, whereas Ag–O bonds were more constrained (σ^2^ = 0.016–0.020 Å^2^) (Figures S5–S7). In all three systems, the unconstrained σ^2^(*T*) fit strategy delivered low R-factors (<0.036) and χ^2^ values close to unity, demonstrating good agreement with the experimental data. Full fitting results for the remaining structural parameters (*N*, Δ*R*, Δ*E*_0_) are given in Tables S2–S4 in the Supporting Information.

In an attempt to disentangle thermal vibrational disorder from static contributions, the correlated Einstein model (Equation 2) was applied next, using the Einstein temperature (*θ_E_*) as a proxy for bond stiffness, expressed as $k=\mu (\frac{2\pi k_{B}\theta_{E}}{h}{)}^{2}$ , (Tables S5–S7). The recalculated temperature-dependent σ^2^(*T*, *θ_E_*) values are shown in Figure 4d–f (open symbols). The Ag_np_/Al_2_O_3_ data set was properly reproduced over the full temperature range (Figure S8), yielding a *θ_E_* = 153 K, consistent with the soft, delocalized metallic bonding characteristic of Ag crystals (Table S6).^84^ However, for the Ag_1_–WO_*x*_/Al_2_O_3_ and Ag_clus_/LTA catalysts, the model failed to accurately reproduce the σ^2^(T) behavior, particularly at temperatures below 573 K (Figures S9 and S10). In the case of Ag_1_–WO_*x*_/Al_2_O_3_, the fit resulted in an unphysically high χ^2^ = 4.13 (Table S7). For Ag_clus_/LTA, the Ag–Ag path delivered a reasonable *θ_E_* = 132 K, aligned with reported values for small clusters and nanoparticles,^84,85^ but the anomalously high *θ_E_* = 347 K obtained for the Ag–O bonds (Table S8) suggests that the correlated Einstein model fails to account for host–guest interactions and complex vibrational coupling within the LTA framework.^76^

This discrepancy highlights the limited applicability of the correlated Einstein model for describing thermal disorder in catalysts featuring subnanometer-sized metal species, such as few-atom nanoclusters and single atoms. In these systems, where the local bonding environment is highly heterogeneous, a single Einstein temperature may not accurately represent the full spectrum of vibrational modes and instead provides only an average description of the lattice dynamics. In practice, different scattering paths often require different effective Einstein or Debye temperatures; therefore, a simple harmonic model may overlook anisotropic motions and multifrequency coupling vibrations.^86^ These findings advise caution in using this classical model to describe the temperature-resolved EXAFS signals during *operando* XAS studies of supported catalyst materials with low-atomicity metal species.

Given the inability of the correlated Einstein model to reproduce the σ^2^(*T*) behavior of Ag_1_–WO_*x*_/Al_2_O_3_ and Ag_clus_/LTA, a complementary linear parametrization was employed to better capture the experimental trends. To assess the sensitivity of vibrational disorder to temperature, σ^2^(*T*) was expressed as σ^2^(*T*) = *αT* + *β*, with *α* and *β* as fitting parameters (eq 3in the Methods section, Figure 4g–i). This approach, justified by the near-linear σ^2^(*T*) dependence observed above room temperature, offers a model-agnostic description free from the single-frequency constraint of the Einstein formalism.

This strategy achieved excellent agreement with the temperature-resolved spectra (Figures S11–S13), delivering low R-factors (<0.036) and χ^2^ values close to unity for the three catalysts, confirming the robustness of the fitting approach. This method also enables a straightforward comparison of thermal response across materials via the slope *α* = dσ^2^/dT. Ag_np_/Al_2_O_3_ showed the steepest slope (*α*_Ag–Ag_ = 3.5 × 10^–5^ Å^2^ ·K^–1^), consistent with the high vibrational disorder sensitivity of large metal nanoparticles to temperature, as inferred from the low *θ_E_* (Table S9). The extracted *α*_Ag–Ag_ closely matched the value inferred from the Einstein temperature, *α*_*θE*_ = 3.8 × 10^–5^ Å^2^ ·K^–1^ (9.7% difference). This agreement validates the linear approximation in the classical regime (*T* ≫ *θ*_*E*=153 K_). Ag_1_–WO_*x*_/Al_2_O_3_ exhibited a lower slope (*α*_Ag–O_ = 2.1 × 10^–5^ Å^2^ ·K^–1^), making it comparatively less sensitive to vibrational disorder due to temperature effects (Table S10). For Ag_clus_/LTA, Ag–O displayed the smallest slope (*α*_Ag–O_ = 1.8 × 10^–5^ Å^2^ ·K^–1^), indicative of framework-imposed rigidity, while intracluster Ag–Ag bonds showed higher sensitivity (*α*_Ag–Ag_ = 2.1 × 10^–5^ Å^2^ ·K^–1^), consistent with a certain degree of structural flexibility of Ag_6_ clusters inside the zeolite cavities (Table S11).

The impact of each fitting model on the statistical coupling between the Debye–Waller factor (σ^2^) and coordination number (*N*) was diagnosed through the correlation matrices reported in Figures S14–S16. These matrices quantify how the uncertainty in one fitted parameter propagates into another, revealing interdependencies that can compromise the physical interpretability of the fit. A high correlation magnitude (|r| close to ± 1) indicates that two parameters are partially redundant or compensate for each other (an increase in one can be offset by a corresponding change in the other) to achieve an apparently good least-squares agreement, but leading to unreliable physical solutions. Minimizing these correlations is, therefore, essential for achieving statistically robust and chemically meaningful EXAFS refinements.

For the unconstrained σ^2^(*T*) model, where σ^2^ floated independently at each temperature, the correlation matrices revealed moderate to strong positive correlations between *N* and σ^2^ (|r| = 0.49–0.84). This behavior reflects the classical amplitude–disorder degeneracy in EXAFS, wherein variations in coordination number can be compensated by ambiguous adjustments in thermal disorder or vice versa. Constraining σ^2^(*T*) using the correlated Einstein model significantly reduced the number of free parameters, replacing multiple σ^2^ terms with a single Einstein temperature (*θ_E_*), but at the cost of introducing strong negative correlations between *N* and *θ_E_* (|r| = 0.87–0.91). This anticorrelation suggests that, within the single-frequency harmonic approximation, the model compensates for changes in amplitude by adjusting *N* and *θ_E_* in opposite directions, likely reflecting a mismatch between the assumed vibrational model and the actual system dynamics.

By contrast, the linear σ^2^(*T*; *α*, *β*) parametrization effectively mitigated these coupling effects. Correlations between *N* and the disorder parameter (slope *α*) dropped around 0.01 < |r| < 0.24, indicating that the model accurately captured the thermal evolution of σ^2^ without overfitting. Although a higher correlation was observed between *N* and the intercept *β* (0.18 < |r| < 0.74), this is largely inconsequential, as *β* acts merely as a baseline offset of the linear trend and lacks independent physical meaning. Overall, the reduction in *N*–σ^2^ correlation under the linear model demonstrates that, within the temperature range investigated, this parametrization provides greater statistical orthogonality and physical transparency for describing thermal disorder in the model Ag-based catalysts, while maintaining excellent agreement with experimental data.

## Conclusions

The convolution of static structural heterogeneity and thermal vibrations, both encoded in the Debye–Waller factor (σ^2^), is of central significance for accurately tracking structural changes in finely dispersed functional metal species, such as supported metal catalysts, from their EXAFS signatures. At those elevated temperatures typical of *in situ* and *operando* studies, this coupling can mask genuine structural changes and, thus, obscure the true structural dynamism of the active metal sites. Across model supported Ag catalysts precisely synthesized to span different degrees of metal aggregation—from conventional supported nanocrystals to subnanometer few-atom ensembles and single atoms—there is a pronounced interplay between dynamic (thermal) and static (structural) disorder contributions to σ^2^.

The correlated Einstein model enables a physically grounded deconvolution of static and thermal contributions, and the extraction of bond-specific force constants. However, a linear parametrization of σ^2^(*T*) provides a concise, empirical description over the relevant experimental temperature range and is particularly useful when bond anharmonicity or vibrational mode mixing violates the assumptions of a purely harmonic oscillator, as is often the case in low-nuclearity species containing only a few or a single metal atom.

In supported Ag nanocrystals, low static disorder combined with relatively soft Ag–Ag bonds leads to Debye–Waller factors dominated by dynamic disorder at elevated temperatures, reflecting largely metal-dominated vibrational behavior, given the limited influence of the chemically inert, low-surface-area α-Al_2_O_3_ support on Ag–Ag bond vibrations. In contrast, in Ag_1_–WO_*x*_/Al_2_O_3_, the WO_*x*_ overlayer provides strong Ag–O anchoring sites that attenuate thermal vibrations in single-atom sites, so that structural heterogeneity becomes the main contributor to σ^2^. For zeolite-confined few-atom Ag clusters, Ag–O bonds to the rigid aluminosilicate framework impose the strongest confinement-induced stabilization, manifested by the highest Einstein temperatures and the shallowest σ^2^(*T*) slopes, while the corresponding intracluster Ag–Ag bonds remain comparatively relaxed and more dynamically responsive. Therefore, our results illustrate how a deconvoluted analysis of σ^2^(*T*) in few-atom, supported metal catalysts is instrumental to rationalize the significant function of specific support materials, e.g., microporous hosts, not only in stabilizing specific metal nuclearities but also in modulating bond stiffness and vibrational dynamics through the imposition of distinct local coordination environments.

Overall, these findings highlight the critical role of local coordination and bond strength in dictating the vibrational dynamics and thermal response of supported metal species. They also underscore the need for tailored analytical strategies, balancing physical rigor with empirical flexibility, to accurately describe disorder when EXAFS is used to characterize solid catalysts and other functional materials featuring (sub)­nanometric metals under variable-temperature conditions. The systematic methodology presented in this work for interpreting temperature-dependent EXAFS data is fully applicable across relevant environments and is expected to broadly support the community in *operando* structural characterization under catalyst activation, reaction, and regeneration treatments.

## Methods

### Synthesis of Supported Ag Nanoparticles (Ag_np_/Al_2_O_3_)

Ag nanoparticles were synthesized on an α-Al_2_O_3_ support by wet impregnation using a precursor solution of silver oxalate (Ag_2_C_2_O_4_). The use of chemically inert and low-surface-area (5.8 m^2^ ·g^–1^) α-Al_2_O_3_ as the carrier material promotes the aggregation of silver into comparatively large nanoparticles. First, Ag_2_C_2_O_4_ was obtained via precipitation of silver nitrate (AgNO_3_, Sigma-Aldrich, ≥99.0%) and oxalic acid (H_2_C_2_O_4_, Sigma-Aldrich, ≥99.0%) in Milli-Q water (AgNO_3_:H_2_C_2_O_4_ molar ratio of 0.38:1). After stirring for 10 min at room temperature, the precipitate was filtered, washed with 1 L of Milli-Q water, and dried overnight under vacuum at room temperature. The final material was recovered and stored under an inert atmosphere.

The α-Al_2_O_3_ support was achieved by calcining γ-Al_2_O_3_ spheres (SASOL, Alumina Spheres 1.8/210), which were crushed and sieved to a size range of 600–800 μm. The spheres were calcined at a heating rate of 3 K·min^–1^ to 1413 K, followed by an isothermal dwell of 4 h at the same temperature.

Silver was incorporated over the α-Al_2_O_3_ support by wet impregnation with an aqueous solution of Ag_2_C_2_O_4_ and ethylenediamine (Ag_2_C_2_O_4_:C_2_H_8_N_2_ molar ratio of 1:3) to attain a nominal loading of 5 wt % Ag on the catalyst. The solvent was removed by evaporation at 333 K under vacuum, and the resulting material was calcined under a N_2_ flow (200 mL·min^–1^) at 723 K (2 K·min^–1^) for 4 h.

### Synthesis of Ag Single-Atom Catalyst (Ag_1_-WO_*x*_/Al_2_O_3_)

A catalyst featuring atomic Ag dispersion was synthesized by oxidative silver redispersion and atom-trapping^87^ onto a polytungstate (WO_*x*_) overlay supported on mesoporous γ-Al_2_O_3_. First, the mesoporous γ-Al_2_O_3_ carrier was dried and degassed under dynamic vacuum (10 mbar) at 423 K for 3 h. The WO_*x*_ overlay was then deposited under stagnant vacuum via incipient wetness impregnation with an aqueous solution of ammonium metatungstate ((NH_4_)_6_H_2_W_12_O_40_, Sigma-Aldrich, 99.99%) with the appropriate salt concentration to obtain a monolayer surface density of ca. 4.5 W_at_ nm^–2^.^88^ The volume of the impregnating solution applied was equivalent to 90% of the total pore volume of the support material, as determined by N_2_ physisorption (0.82 cm^3^·g^–1^). The impregnated solid was calcined at 973 K (3 K·min^–1^) for 4 h in a quartz plug flow reactor operated with an airflow rate of 80 mL·min^–1^ per gram of material.

After cooling, the material was transferred into a round-bottom flask containing a solution of silver acetylacetonate (Ag­(acac), Sigma-Aldrich, 98%) in acetone and kept under constant stirring (200 rpm) for 2 h. The amounts of both Ag­(acac) and WO_*x*_/γ-Al_2_O_3_ support were determined to attain a desired Ag coverage of ca. 0.8 Ag_at_·nm^–2^. Subsequently, the solvent was removed using a rotary evaporator operating at 200 mbar and 333 K. After this step, the sample was dried overnight at 373 K and then calcined at 973 K (3 K·min^–1^) for 4 h in a muffle furnace under stagnant air to achieve oxidative redispersion of the single Ag atoms. The final Ag concentration in the Ag_1_–WO_*x*_/Al_2_O_3_ catalyst was 1.63 wt %, as determined by scanning electron microscopy with energy-dispersive X-ray analysis (SEM-EDX).

### Synthesis of Zeolite-Confined Ag Clusters (Ag_clus_/LTA)

Ag subnanometric clusters were developed within the sodalite cages of a Linde-type A (LTA) zeolite carrier via ion exchange of Na-LTA synthesized as detailed elsewhere.^89^ 0.5 g of Na-LTA zeolite was added to 250 mL of an aqueous solution of silver nitrate (AgNO_3_, 99%, Sigma-Aldrich) contained in a polypropylene bottle. The stoichiometric concentration of Ag in the solution was equivalent to 1/12 of the Na ion content of the pristine zeolite (9.88 Ag wt %) as determined by ICP-OES. The ion-exchange was conducted at room temperature under constant stirring (250 rpm) for 16 h. The exchanged solid was recovered by filtration and dried in a two-step treatment first at 353 K (1 K·min^–1^) for 1 h and then at 383 K (1 K·min^–1^) for 1 h, to prevent the collapse of the zeolitic framework due to fast water removal. Finally, the catalyst was activated in the same muffle oven at 723 K (3 K·min^–1^) for 2 h under stagnant air. In every synthesis step, the sample was handled under dark room conditions to avoid the inherent photoreduction of silver species. The final Ag concentration in Ag_clus_/LTA was 4.2 wt %, as determined by ICP-OES.

## Characterization Methods

### Inductively Coupled Plasma Optical Emission Spectrometry (ICP-OES)

The metal content of Ag_np_/Al_2_O_3_ and Ag_clus_/LTA catalysts was quantified by Inductively Coupled Plasma Optical Emission Spectrometry (ICP-OES). The samples were analyzed using an iCAP^TM^ PRO after disaggregating 30 mg of the sample in an HNO_3_/HF solution at room temperature. Quantification was based on calibration lines derived from the analysis of certified standards (CertiPUR) within the expected concentration ranges for Ag, Na, Si, Al.

### Aberration-Corrected High-Angle Annular Dark-Field (HAADF) and Integrated Differential Phase Contrast (iDPC) Scanning Transmission Electron Microscopy (STEM)

HAADF-STEM imaging was performed in a double (spherical­(C_s_) and chromatic (C_c_)) aberration-corrected Thermo Fisher Scientific Spectra 300 (S)­TEM microscope equipped with a monochromator. In both instances, the microscope was operated at an acceleration voltage of 300 kV. Powder samples were directly cast on Cu and Au grids coated with a holey carbon film prior to observation. For Ag_1_–WO_*x*_/Al_2_O_3_, the difference in the atomic number between W atoms (Z_W_ = 74) and Ag atoms (Z_Ag_ = 47) enabled element-sensitive intensity analysis of 3D Z-contrast maps in micrograph regions containing Ag atoms associated with polytungstate WO_*x*_ clusters. Integrated differential phase contrast (iDPC) imaging of Ag_clus_/LTA was conducted on the double-corrected Thermo Fisher Scientific Spectra 300 microscope operated at 60 kV. The STEM beam was monochromatized using a TFS Optimono to avoid chromatic aberration while working at low voltage.

### X-ray Diffraction (XRD)

Powder X-ray diffraction patterns were recorded from 10° to 90° (2θ) with a step size of 0.020° (2θ) in a Bragg–Brentano geometry using a PANalytical CUBIX diffractometer equipped with an X’Celerator detector. Cu Kα radiation (λ_1_ = 1.5406 Å, λ_2_ = 1.5444 Å) was used as the incident X-ray source, operating at 45 mA and 40 kV. The length of the goniometer arm was 200 mm, and a fixed divergence slit with an aperture of 1/8° was used.

### X-ray Absorption Spectroscopy (XAS) Experiments

X-ray absorption spectra were recorded at the Ag K edge (25.514 keV) in the NOTOS (BL16) beamline station of the ALBA synchrotron light source (Spain). The beam was monochromatized using a double-crystal monochromator equipped with Si(111) crystals, while higher-order harmonics were filtered using a Rh-coated Si(111) mirror. The measurements were performed in a capillary flow cell customized to operate as a fixed-bed microreactor under representative gas–solid process conditions. A sieve fraction (100–200 μm) of the sample was placed at the center of a quartz capillary and packed between two beds of SiC granules (100–200 μm) to enhance thermal conductivity. The catalyst was heated stepwise from 298 to 723 K with a heating rate of 5 K·min^–1^ by a hot air blower (220 V/1000 W, nozzle Ø 5 mm ID, XDS Oxford Ltd.), positioned 5 mm orthogonal to the capillary to ensure uniform temperature distribution along the packed bed (5 mm length).

The capillary dimensions and gas stream composition were tailored to each sample condition to ensure an appropriate X-ray attenuation coefficient and to maintain the metal aggregation level of Ag in each system, respectively. To prevent the reductive agglomeration of Ag atoms in the Ag_1_–WO_*x*_/Al_2_O_3_ and Ag_clus_/LTA catalysts, a gas stream of 20% O_2_/He (5 mL·min^–1^) was selected. In contrast, a pure He stream (5 mL·min^–1^) was used during the thermal treatment of Ag_np_/Al_2_O_3_ to prevent oxidative disruption of the Ag nanoparticles and metal redispersion at high temperatures.

All EXAFS spectra were collected *in situ* from 298 to 723 K with incremental steps of 50–100 K. Several scans were acquired at each measurement step (allowing 1 h for temperature stabilization) and averaged to improve the signal-to-noise ratio to an optimal level. The spectra of Ag_np_/Al_2_O_3_ and Ag_clus_/LTA were measured in transmission mode using ionization chambers filled with appropriate gases, ensuring photon absorption levels of 15% at *I*_0_ and 80% at *I*_1_. For Ag_1_–WO_*x*_/Al_2_O_3_, where the silver content is significantly lower, EXAFS data were acquired in fluorescence mode by using a 13-element silicon drift detector (SDD), positioned at a 45° angle relative to the capillary, to maximize signal collection and ensure reliable data acquisition.

## EXAFS Data Treatment and Fitting Models

XAS data reduction and extraction of the χ­(*k*) functions were carried out using the ATHENA component of the Demeter software package (v0.9.26).^34^ The raw absorption spectra, *μ*(*E*), were aligned using a reference Ag foil measured simultaneously with the samples. The absorption edge energy (*E*_0_) was determined from the maximum of the first derivative in the XANES region. Subsequently, the pre-edge and postedge backgrounds were subtracted, and the spectra were normalized to unity beyond the edge to account for differences in sample thickness and concentration. The EXAFS oscillations were converted to reciprocal space χ­(*k*) and *k* ^3^-weighted to enhance the contributions from high-*k* regions. A Fourier transform was applied over 1.0 < χ­(*k*) < 8.0 Å^–1^ using a Hanning window to obtain χ­(R) in real space.

EXAFS fitting and structural parameter refinement were performed in the ARTEMIS component of Demeter, employing theoretical phase and amplitude functions generated by the FEFF6 code^90^ based on crystallographic structures of reference compounds: Ag (*Fm3m*), Ag_2_WO_4_ (*Pnnm*), and Ag_2_O (*Pn3m*). The χ­(*k*) spectra were modeled according to the standard EXAFS equation.^36^

1 $$\chi \left(k\right)=\underset{j}{\sum }\frac{N_{j}S_{0}^{2}}{kR_{j}^{2}}f_{j}\left(k\right)e^{-2k^{2}\sigma_{j}^{2}}e^{-\frac{2R_{j}}{\lambda \left(k\right)}}\sin\left[2kR_{j}+\delta_{j}\left(k\right)\right]$$

where *k* is the photoelectron wave vector, *N_j_* is the coordination number of the j-th shell, $S_{0}^{2}$ is the amplitude reduction factor accounting for many-body effects, *f_j_* (*k*) is the effective scattering amplitude of neighboring atoms, λ­(*k*) is the photoelectron mean free path, and *δ*_*j*_(*k*) is the phase shift experienced by the backscattered photoelectron.

The term sin­[2*kR_j_* + *δ_j_* (*k*)]­gives rise to the characteristic oscillations of χ­(*k*), whose frequency depends on the absorber–scatterer distance, *R_j_*. The amplitude of χ­(*k*) increases with the number of neighboring atoms, *N*_*j*_, but is attenuated by two exponential damping terms: $e^{-\frac{2R_{j}}{\lambda \left(k\right)}}$ , accounting for inelastic losses along the photoelectron trajectory, and $e^{-2k^{2}\sigma_{j}^{2}}$ , which incorporates the Debye–Waller factor (σ^2^), reflecting the loss of coherence between the outgoing and backscattered waves due to thermal vibrations and structural disorder. The scattering amplitude *f_j_* (*k*) and phase shift *δ_j_* (*k*) contain chemical and structural specificity, thereby enabling the discrimination of neighboring atoms and their local environments.

To resolve the temperature dependence of the Debye–Waller factor σ^2^(*T*) and assess the impact of local structural disorder on the EXAFS signal, three complementary parametrization strategies were applied:

### Unconstrained σ^2^(T) Fitting

In the first approach, the Debye–Waller factor σ^2^ was treated as an independent free parameter for each temperature. Coordination numbers (*N_j_*) and interatomic distance shifts (Δ*R_j_*) were kept constant across temperatures and optimized according to the absorber–scatterer pair (Ag–Ag, Ag–O) to reflect the distinct local environments. The energy shift Δ*E*_0_ was maintained as a global parameter across the data set. This unconstrained model provides direct estimates of σ^2^ across the temperature range, entangling both structural and thermal disorder effects.

### Correlated Einstein Model σ^2^(*T*; *θ_E_*) Fitting

To resolve the dynamic and static contributions to disorder, σ^2^(*T*) was described using the correlated Einstein model.^46^

2 $$\sigma_{j}^{2}\left(T;\theta_{E}\right)=\frac{\hbar }{2\mu \omega_{E}}\coth\left(\frac{\hbar \omega_{E}}{2k_{B}T}\right)=\frac{\hbar^{2}}{2\mu k_{B}\theta_{E}}\coth\left(\frac{\theta_{E}}{2T}\right)$$

where ℏ = 1.055 × 10^–34^ J·s denotes the reduced Planck constant, *ω_E_* = *K*_B_ *θ*_E_/ℏ, defines the Einstein frequency in terms of the Einstein temperature *θ_E_*, *k_B_* = 1.38 × 10^–23^ J·K^–1^ is the Boltzmann constant, $\mu =\frac{m_{1}m_{2}}{m_{1}+m_{2}}$ is the reduced mass of the atomic pair, and *T* is the temperature of the sample.

In this model, the σ^2^(*T*) values were constrained by using Equation 2, with the Einstein temperature (*θ_E_*) treated as a free-fitting parameter. Coordination numbers (*N_j_*) and interatomic distance shifts (Δ*R_j_*) were kept constant across temperatures and optimized according to the absorber–scatterer pair (Ag–Ag, Ag–O) to reflect the distinct local environments. The energy shift Δ*E*_0_ was maintained as a global parameter across the dataset.

### Linear Parameterization σ^2^(*T*; *α*, *β*) Fitting

In a third approach, σ^2^(*T*) was modeled as a linear function of the temperature:

3 $$\sigma_{j}^{2}\left(T;\;\alpha ,\beta \right)=\alpha_{j}T+\beta_{j}$$

While *α_j_* provides a scalar descriptor of the bond stiffness via the approximation *α*_*j*_ ≈ h̅^2^/(*μk_B_θ*_E_^2^), enabling direct comparison of vibrational strength across systems, the intercept *β_j_* lacks strict physical meaning. This expression is valid in the high-temperature regime (*T* > *θ_E_*), where the thermal motion becomes nearly classical and the coth function in Equation 2 can be approximated to coth(θE2T)≈2TθE , leading to a direct proportionality between σ^2^ and *T*.^91^ However, at low temperatures approaching absolute zero (*T* → 0 K), the Debye–Waller factor σ^2^ is dominated by quantum effects and largely deviates from linearity.^35^ At the opposite extreme, under sufficiently high temperatures (*T* ≫ *θ_E_*), anharmonic contributions from phonon–phonon interactions may also induce nonlinear deviations, further limiting the validity of the linear approximation.^92^

Here, Equation 3 was applied to define σ^2^(*T*) over the 298–723 K range, with *α_j_* and *β_j_* used as independent free-fitting parameters. Coordination numbers (*N_j_*) and interatomic distance shifts (Δ*R_j_*) were kept constant across temperatures and optimized according to the absorber–scatterer pair (Ag–Ag, Ag–O) to reflect the distinct local environments. The energy shift Δ*E*_0_ was maintained as a global parameter across the dataset.

## Supplementary Material



## Notes

Experimental details on catalytic testing procedures; additional characterization data (HAADF-STEM, XRD, TGA–DSC); temperature-resolved EXAFS spectra and fits; model fitting analyses; correlation matrices of fitting parameters; and complete tabulated structural results (PDF)
