# Supplementary material for: Vibrational Disorder Effects on Temperature-Resolved X‑Ray Absorption Signatures of Metal Catalysts: From Single-Atoms to Clusters and Nanoparticles
Source: ACS Nano. 2026 Apr 27;20(18):13572–85. doi: 10.1021/acsnano.5c20042 (PMC13173657; doi:10.1021/acsnano.5c20042)
Supplement: Supplementary file 1 [file nn5c20042_si_001.pdf]

-Supporting information-

Vibrational disorder effects on temperature-resolved X-ray absorption signatures of metal catalysts: from single-atoms to clusters and nanoparticles

Wilson Henao[1], Ivan López-Luque[1], Gonzalo Prieto[1]*, Giovanni Agostini[2,3]*

[1] ITQ Instituto de Tecnología Química, Universitat Politècnica de València-Consejo Superior de Investigaciones Científicas (UPV-CSIC) Av. Los Naranjos S/N, Valencia, 46022, Spain

[2] ALBA Synchrotron Light Source Carrer de la Llum 2-26, Cerdanyola del Vallès, Barcelona, 08290, Spain

[3] Elettra-Sincrotrone, Basovizza, Trieste, 34149, Italy

* Corresponding authors: prieto@itq.upv.es, giovanni.agostini@elettra.eu

Table of Contents

Supporting Methods

Catalysis testing

Ethylene epoxidation..4

Carboxylation of phenylacetylene with CO_2..4

Supporting Figures

Figure S1. Time-lapsed collection of HAADF-STEM micrographs for the Ag_{clus}/LTA catalyst showing the fast development and progressive growth of Ag nanoclusters within the zeolite framework under cumulative electron beam irradiation 5

Figure S2. Powder X-ray diffraction patterns for the as-synthesized supported silver nanoparticles (Ag_{np}/Al_2O_3), silver clusters (Ag_{clus}/LTA) and silver single-atom (Ag_1-WO_x/Al_2O_3) catalysts 6

Figure S3. Fit of a,b) the Ag K-edge k^3-χ(k) EXAFS spectra and c,d) their corresponding Fourier transforms FT-χ(R) signals for the as-synthesized catalysts and after in situ thermal treatment 6

Figure S4. Thermogravimetric Analysis (TGA) and Differential Scanning Calorimetry (DSC) profiles showing the dehydration behavior of Ag_{clus}/LTA.. 7

Figure S5. Fit of temperature-resolved Ag K-edge EXAFS spectra for the Ag_{np}/Al_2O_3 catalyst using the unconstrained σ^2(T) model from Equation 1 (main text)... 7

Figure S6. Fit of temperature-resolved Ag K-edge EXAFS spectra for the Ag_1-WO_x/Al_2O_3 catalyst using the unconstrained σ^2(T) model from Equation 1 (main text). 8

Figure S7. Fit of temperature-resolved Ag K-edge EXAFS spectra for the Ag_{clus}/LTA catalyst using the unconstrained σ^2(T) model from Equation 1 (main text)... 8

Figure S8. Fit of temperature-resolved Ag K-edge EXAFS spectra for the Ag_{np}/Al_2O_3 catalyst using the correlated Einstein model $\sigma^2(T; \theta_E)$ from Equation 2 (main text) 9

Figure S9. Fit of temperature-resolved Ag K-edge EXAFS spectra for the Ag_1-WO_x/Al_2O_3 catalyst using the correlated Einstein model $\sigma^2(T; \theta_E)$ from Equation 2 (main text) 9

Figure S10. Fit of temperature-resolved Ag K-edge EXAFS spectra for the Ag_{clus}/LTA catalyst using the correlated Einstein model to $\sigma^2(T; \theta_E)$ from Equation 2 (main text)... 10

Figure S11. Fit of temperature-resolved Ag K-edge EXAFS spectra for the Ag_{np}/Al_2O_3 catalyst using the linear parameterization $\sigma^2(T; \alpha, \beta)$ from Equation 3 (main text)... 10

Figure S12. Fit of temperature-resolved Ag K-edge EXAFS spectra for the Ag_1-WO_x/Al_2O_3 catalyst using the linear parameterization $\sigma^2(T; \alpha, \beta)$ from Equation 3 (main text)... 11

Figure S13. Fit of temperature-resolved Ag K-edge EXAFS spectra for the Ag_{clus}/LTA catalyst using the linear parameterization $\sigma^2(T; \alpha, \beta)$ from Equation 3 (main text)... 11

Figure S14. Correlation matrices of the fitting parameters obtained from the temperature-resolved EXAFS analysis of the Ag_{np}/Al_2O_3 catalyst using three fitting approaches 12

Figure S15. Correlation matrices of the fitting parameters obtained from the temperature-resolved EXAFS analysis of the Ag_1-WO_x/Al_2O_3 catalyst using three fitting approaches 13

Figure S16. Correlation matrices of the fitting parameters obtained from the temperature-resolved EXAFS analysis of the Ag_{clus}/LTA catalyst using three fitting approaches.. 14

Supporting Tables

Table S1. Summary of the EXAFS fitting parameters for the as-synthesized model catalysts and after the thermal treatment 15

Table S2. Model catalytic reactions performed over Ag_{np}/Al_2O_3 and Ag_1-WO_x/Al_2O_3 catalysts, demonstrating their functional relevance and catalytic performance 15

Table S3. Summary of the structural parameters derived from the fitting of the temperature-resolved Ag K-edge EXAFS signals for the Ag_{np}/Al_2O_3 catalyst using the unconstrained $\sigma^2(T)$ model from Equation 1 (main text)........ 16

Table S4. Summary of the structural parameters derived from the fitting of the temperature-resolved Ag K-edge EXAFS signals for the Ag_1-WO_x/Al_2O_3 catalyst using the unconstrained $\sigma^2(T)$ modelfrom Equation 1 (main text) 16

Table S5. Summary of the structural parameters derived from the fitting of the temperature-resolved EXAFS signals for the Ag_{clus}/LTA catalyst using the unconstrained $\sigma^2(T)$ model from Equation 1 (main text)........ 17

Table S6. Summary of the structural parameters derived from the fitting of the temperature-resolved Ag K-edge EXAFS signals for the Ag_{np}/Al_2O_3 catalyst using the correlated Einstein model $\sigma^2(T; \theta_E)$ from Equation 2 (main text)........ 17

Table S7. Summary of the structural parameters derived from the fitting of the temperature-resolved Ag K-edge EXAFS signals for the Ag_1-WO_x/Al_2O_3 catalyst using the correlated Einstein model $\sigma^2(T; \theta_E)$ from Equation 2 (main text) 18

Table S8. Summary of the structural parameters derived from the fitting of the temperature-resolved EXAFS signals for the Ag_{clus}/LTA catalyst using the correlated Einstein model $\sigma^2(T; \theta_E)$ from Equation 2 (main text)........ 18

Table S9. Summary of the structural parameters derived from the fitting of the temperature-resolved Ag K-edge EXAFS signals for the Ag_{np}/Al_2O_3 catalyst using the linear parameterization $\sigma^2(T; \alpha, \beta)$ from Equation 3 (main text) 19

Table S10. Summary of the structural parameters derived from the fitting of the temperature-resolved EXAFS signals for the Ag_1-WO_x/Al_2O_3 catalyst using the linear parameterization $\sigma^2(T; \alpha, \beta)$ from Equation 3 (main text) 19

Table S11. Summary of the structural parameters derived from the fitting of the temperature-resolved EXAFS signals for the Ag_{clus}/LTA catalyst using the linear parameterization $\sigma^2(T; \alpha, \beta)$ from Equation 3 (main text) 20

Supporting Methods

Catalysis testing

Ethylene epoxidation

Ethylene epoxidation was evaluated over Ag_{np}/Al_2O_3 using a Microactivity Effi-Solo reactor unit (PID Eng&Tech) equipped with a tubular SS316 fixed-bed reactor (9.1 mm i.d.) inserted into an annular electric furnace. The setup provides three gas inlets, each controlled by a high-precision mass flow controller (Bronkhorst High-Tech Instruments).

For catalytic testing, 100 mg of catalyst was sieved to a particle size range of 600–800 µm and diluted with SiC granules (Sigma-Aldrich, ≥97.5%, 600–800 µm) to improve heat transfer within the packed bed (total bed volume: 3.2 mL). The catalyst bed was supported on a quartz wool plug inside a tubular reactor. The catalyst was activated *in situ* at 498 K (5 K min^{-1}) under 5% O_2/He (20 mL min^{-1}) at 10 bar for 3 h, followed by a He purge (20 mL min^{-1}) for 1 h. After activation, two feed streams were introduced: 77.8 mL min^{-1} of C_2H_4/Ar/He (5/5/90 vol %) and 22 mL min^{-1} of O_2/He (5/95 vol %). This resulted in a final inlet composition of C_2H_4/O_2/Ar/He (3.89/1.11/3.89/91.11 vol %), corresponding to a $C_2H_4:O_2$ ratio of 3.5. The weight hourly space velocity (WHSV) was 2.9 gC_2H_4 gcat^{-1} h^{-1}. The reaction was kept at 498 K and 10 bar for 40 h, until steady-state conversion was reached.

Reaction products were quantified online using an Agilent 8890 GC equipped with two analytical channels. The first channel (CP-Sil 5 CB + MolSieve 5A capillary columns) was coupled to a TCD for permanent gases and light compounds (He, H_2, O_2, CH_4, CO, CO_2, H_2O, and C_2 hydrocarbons). The second channel (CP-Sil 5 CB) was connected to an FID for hydrocarbons (C_1-C_{10}) and oxygenates (C_1-C_6). A PolyARC unit installed upstream of the FID converted carbon-containing species to CH_4 via combustion (to CO_2) and subsequent hydrogenation, enabling a near-unity carbon response factor. Conversions and yields were determined from calibrated peak areas.

Carboxylation of phenylacetylene with CO_2

Ag_1-WO_x/Al_2O_3 was tested in the carboxylation of phenylacetylene with CO_2 using a high-throughput experimentation (HTE) stainless-steel autoclave reactor equipped with inner 8 mL glass vials. 16.6 mg of catalyst (2.0 mol% relative to phenylacetylene) were combined with cesium carbonate (2.0 equivalents) as a base promoter. Phenylacetylene (0.25 mmol) was subsequently added, followed by anhydrous dimethylformamide (DMF) to achieve a total reaction volume of 2.0 mL.

The reaction mixture was prepared under an inert nitrogen atmosphere using standard glovebox techniques. Each vial was equipped with an individual magnetic stir bar to ensure homogeneous mixing of the slurry-phase reaction (900 rpm). The vials were partially sealed and rated for operation under moderate pressure prior to removal from the glovebox. The assembled autoclave was connected to a vacuum pump to evacuate residual nitrogen and then positioned in a temperature-controlled heating block. The system was heated to 313 K and subsequently pressurized with CO_2 (5 bar). The reaction was maintained under these conditions for 45 min.

Reaction products were quantified by high-performance liquid chromatography (HPLC) using a UV detector (Agilent). An aliquot of the crude reaction mixture was diluted in a biphenyl/acetonitrile solution (as internal standard), followed by filtration through a 0.45 µm nylon membrane prior to injection. Chromatographic separation was achieved on a reversed-phase C18 column. The mobile phase consisted of water (pH = 3, adjusted with trifluoroacetic acid) and acetonitrile under gradient conditions optimized to ensure baseline separation of phenylacetylene, phenylpropiolate, and biphenyl (ISTD). The flow rate was set to 1.0 mL min^{-1}, and UV detection was performed at 245 and 256 nm. Quantification was carried out using external calibration curves constructed from independently prepared standards, ensuring linear response within the concentration range investigated. Conversion and yield values were calculated from calibrated peak areas.

Supporting Figures

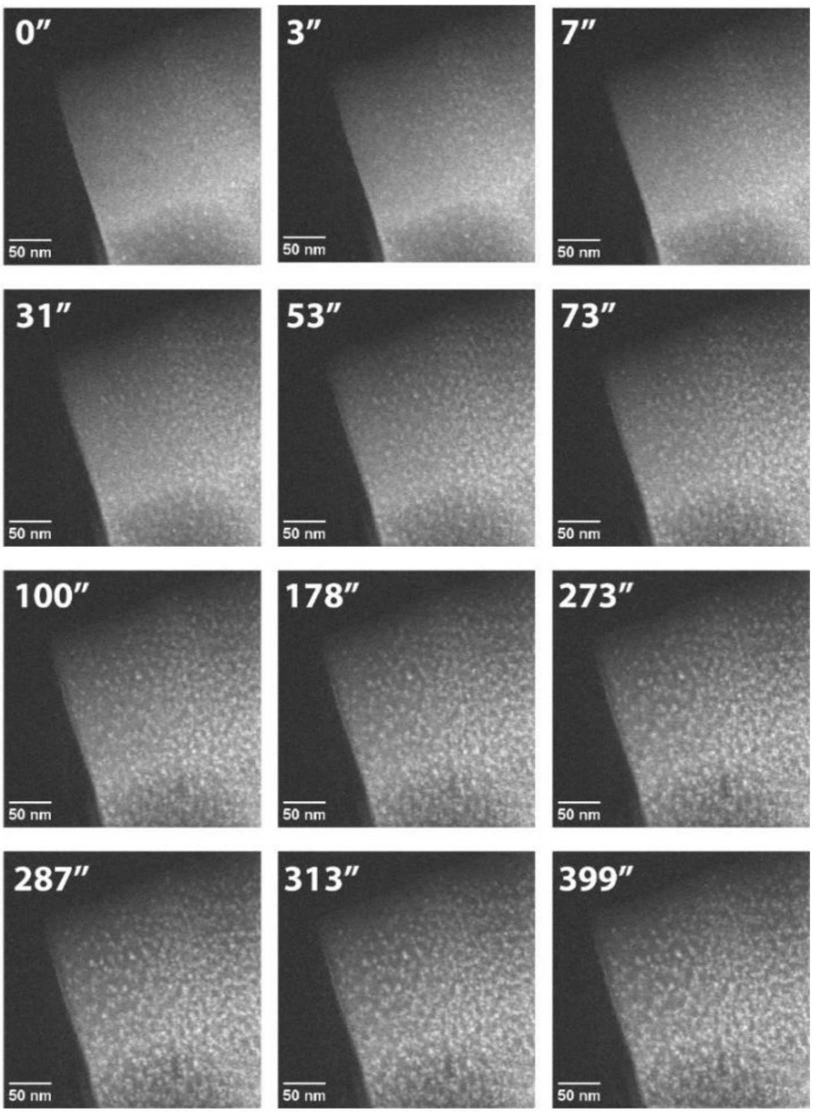

Figure S1. Time-lapsed collection of HAADF-STEM micrographs for the Ag_{clus}/LTA catalyst showing the fast development and progressive growth of Ag nanoclusters within the zeolite framework under cumulative electron beam irradiation. Pristine few-atom Ag clusters could not be directly imaged due to the high electron-beam sensitivity of the material.

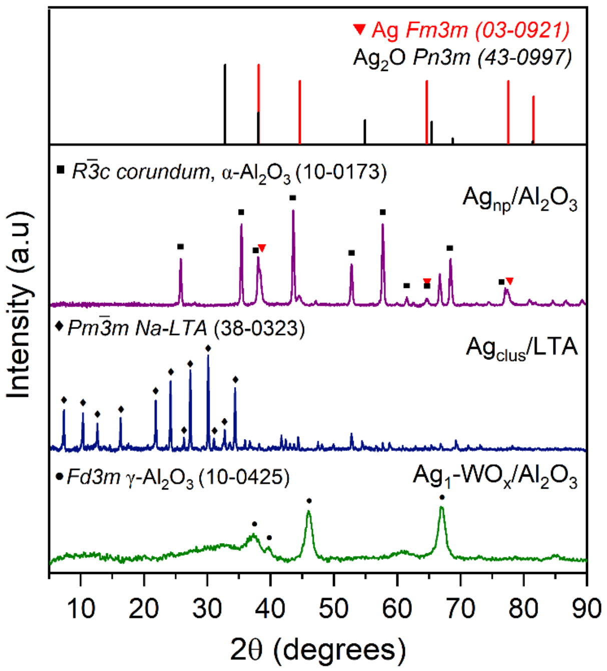

Figure S2. Powder X-ray diffraction patterns for the as-synthesized supported silver nanoparticles (Ag_{np}/Al_2O_3), silver clusters (Ag_{clus}/LTA), and silver single-atom (Ag_1-WO_x/Al_2O_3) catalysts. The reference diffraction peaks of their respective supports α-Al_2O_3, LTA zeolite, and γ-Al_2O_3 together with reference diffraction patterns of Ag_2O and metallic Ag phases are included for indexing purposes.

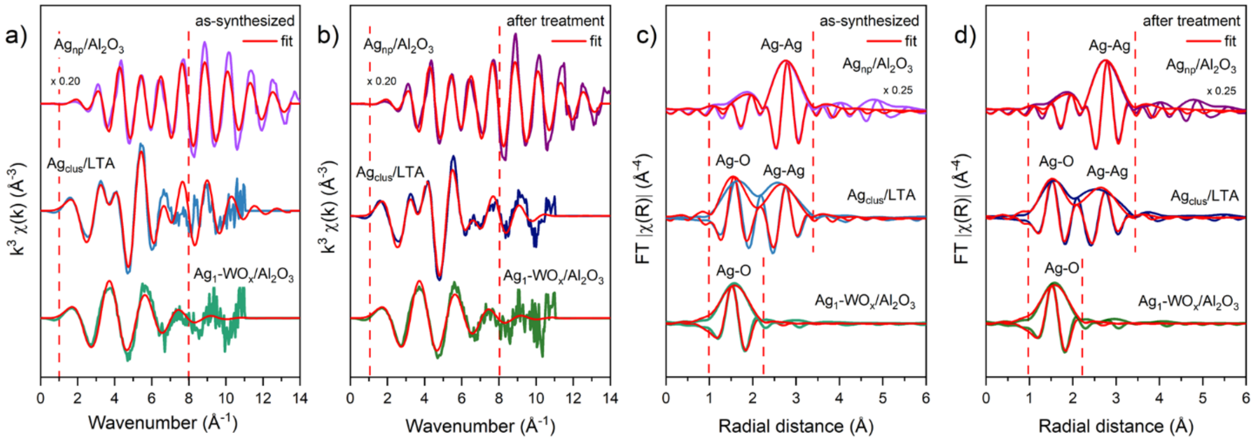

Figure S3. Fit of the Ag K-edge k^3-weighted χ(k) EXAFS spectra in panels a,b) and c,d) their corresponding Fourier transforms FT-|χ(R)| signals (uncorrected for phase shift) for the catalysts in the as-synthesized state and after in situ thermal treatment. Red solid lines represent the fitted spectra. The k-range defined for the Fourier transform and the R-range used for fitting are indicated by red dashed lines. Structural parameters derived from the fitting are summarized in **Table S1**.

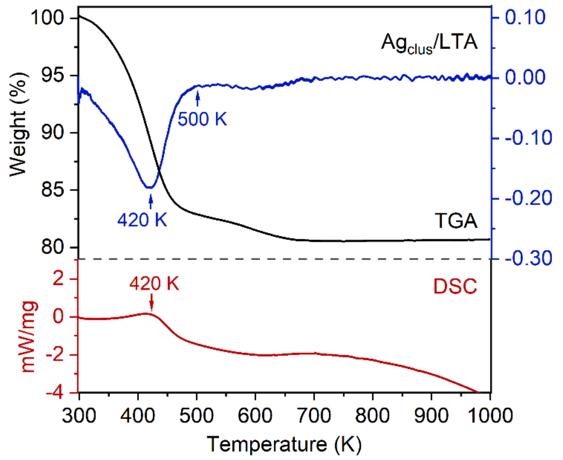

Figure S4. Thermogravimetric Analysis (TGA) and Differential Scanning Calorimetry (DSC) profiles showing the dehydration behavior of Ag_{clus}/LTA. The sample (~10 mg) was heated from 298 K to 1000 K (5 K min^{-1}) in a STA 449 F3 Jupiter thermal analyzer (NETZSCH) under a synthetic air flow (20% O_2/N_2, 10 mL min^{-1}).

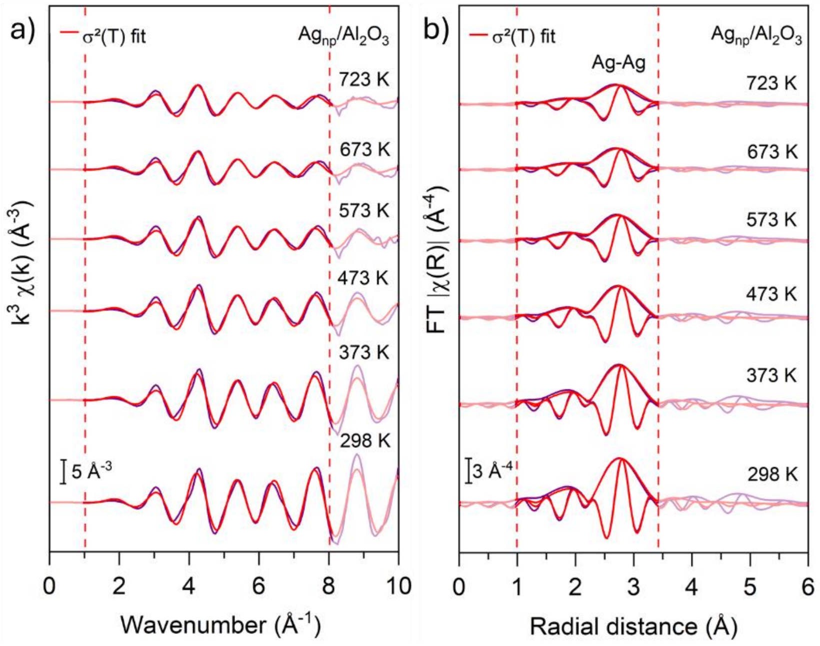

Figure S5. Fit of temperature-resolved Ag K-edge EXAFS spectra for the Ag_{np}/Al_2O_3 catalyst using the unconstrained $\sigma^2(T)$ model from Equation 1 (main text). a) k^3-weighted $\chi(k)$ EXAFS spectra in the reciprocal k-space and b) their corresponding Fourier transforms FT-$|\chi(R)|$ (uncorrected for phase shift). Red solid lines represent the fitted spectra. The k-range defined for the Fourier transform and the R-range used for fitting are indicated by red dashed lines. Structural parameters derived from the fitting are summarized in **Table S3**.

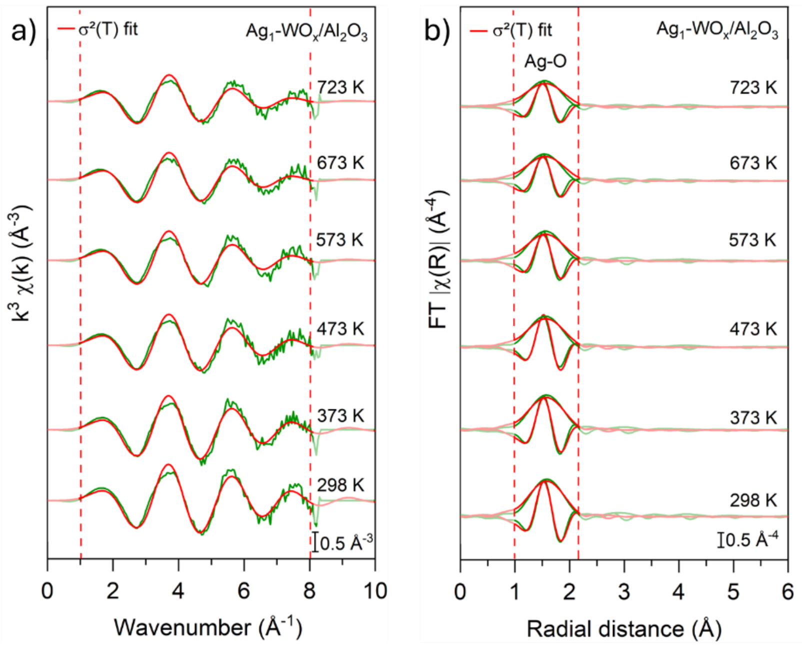

Figure S6. Fit of temperature-resolved Ag K-edge EXAFS spectra for the Ag_1-WO_x/Al_2O_3 catalyst using the unconstrained σ²(T) model from Equation 1 (main text). a) k^3-weighted χ(k) EXAFS spectra in the reciprocal k-space and b) their corresponding Fourier transforms FT-|χ(R)| (uncorrected for phase shift). Red solid lines represent the fitted spectra. The k-range defined for the Fourier transform and the R-range used for fitting are indicated by red dashed lines. Structural parameters derived from the fitting are summarized in **Table S4**.

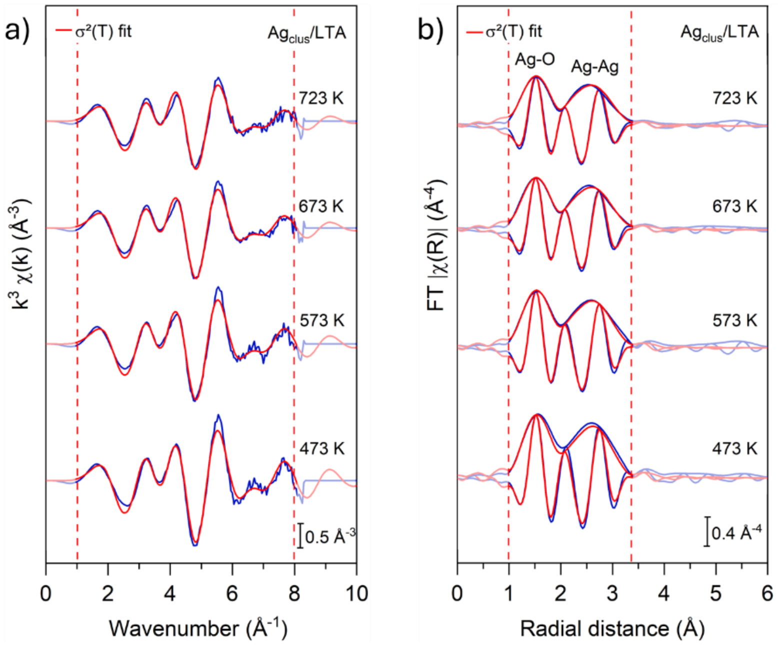

Figure S7. Fit of temperature-resolved Ag K-edge EXAFS spectra for the Ag_{clus}/LTA catalyst using the unconstrained σ²(T) model from Equation 1 (main text). a) k^3-weighted χ(k) EXAFS spectra in the reciprocal k-space and b) their corresponding Fourier transforms FT-|χ(R)| (uncorrected for phase shift). Red solid lines represent the fitted spectra. The k-range defined for the Fourier transform and the R-range used for fitting are indicated by red dashed lines. Structural parameters derived from the fitting are summarized in **Table S5**.

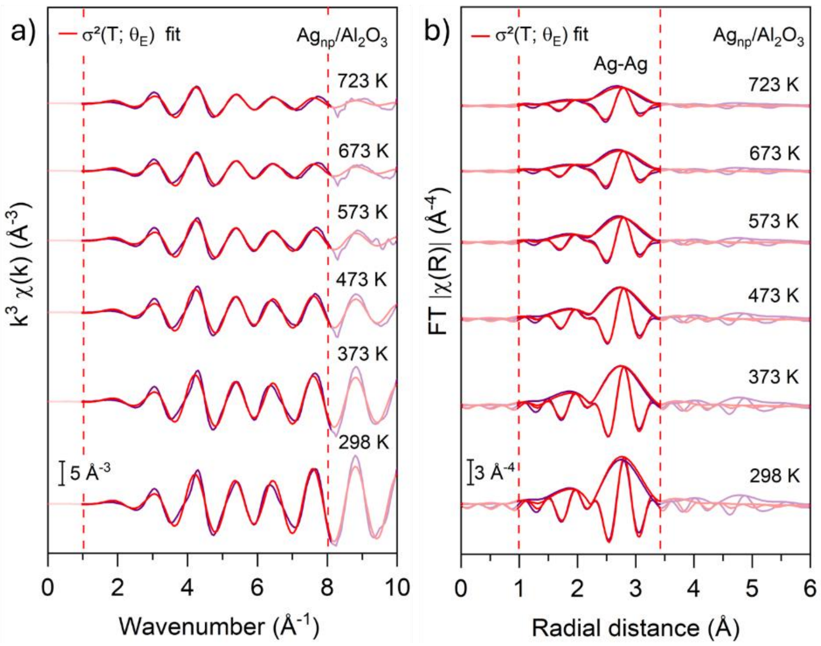

Figure S8. Fit of temperature-resolved Ag K-edge EXAFS spectra for the Ag_{np}/Al_2O_3 catalyst using the correlated Einstein model σ^2 (T; θ_E) from Equation 2 (main text). a) k^3-weighted χ(k) EXAFS spectra in the reciprocal k-space and b) their corresponding Fourier transforms FT-|χ(R)| (uncorrected for phase shift). Red solid lines represent the fitted spectra. The k-range defined for the Fourier transform and the R-range used for fitting are indicated by red dashed lines. Structural parameters derived from the fitting are summarized in **Table S6**.

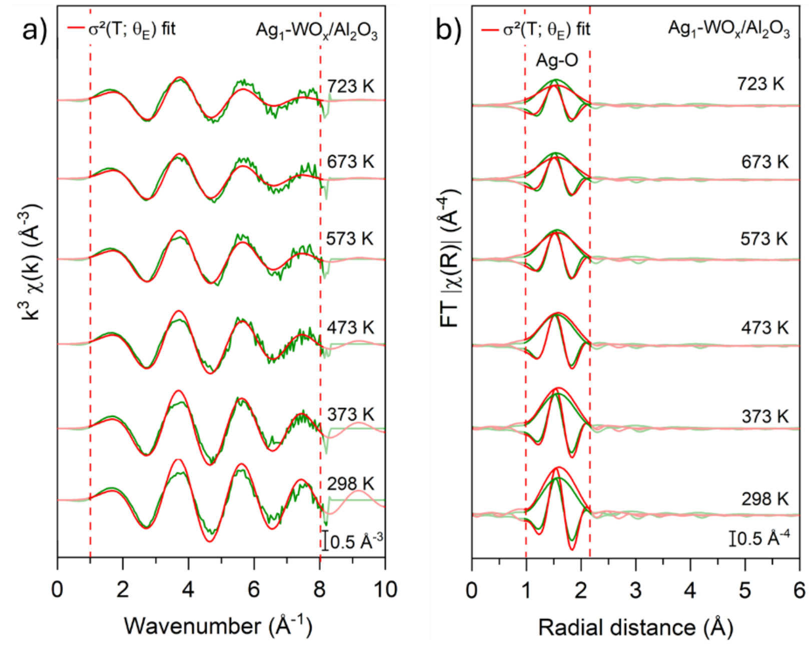

Figure S9. Fit of temperature-resolved Ag K-edge EXAFS spectra for the Ag_1-WO_x/Al_2O_3 catalyst using the correlated Einstein model σ^2 (T; θ_E) from Equation 2 (main text). a) k^3-weighted χ(k) EXAFS spectra in the reciprocal k-space and b) their corresponding Fourier transforms FT-|χ(R)| (uncorrected for phase shift). Red solid lines represent the fitted spectra. The k-range defined for the Fourier transform and the R-range used for fitting are indicated by red dashed lines. Structural parameters derived from the fitting are summarized in **Table S7**.

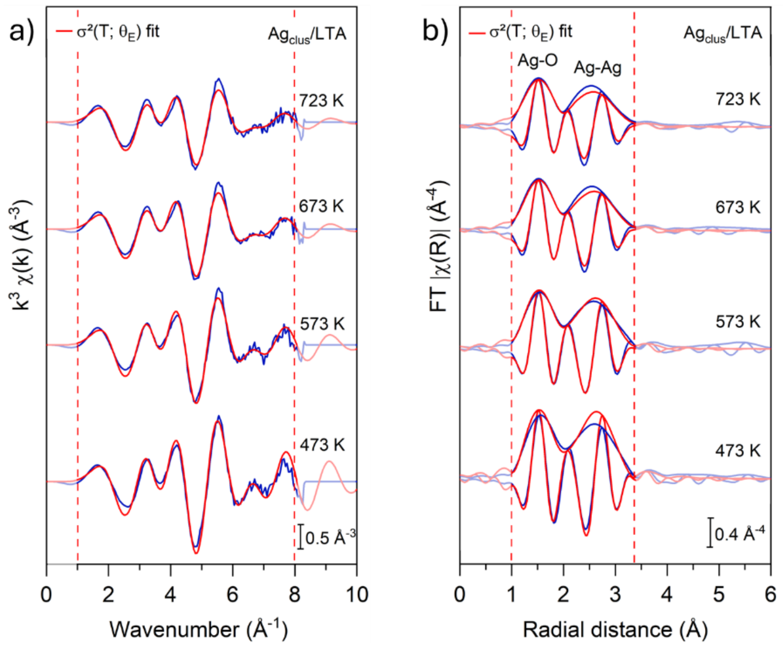

Figure S10. Fit of temperature-resolved Ag K-edge EXAFS spectra for the Ag_{clus}/LTA catalyst using the correlated Einstein model σ^2 (T; θ_E) from Equation 2 (main text). a) k^3-weighted χ(k) EXAFS spectra in the reciprocal k-space and b) their corresponding Fourier transforms FT-|χ(R)| (uncorrected for phase shift). Red solid lines represent the fitted spectra. The k-range defined for the Fourier transform and the R-range used for fitting are indicated by red dashed lines. Structural parameters derived from the fitting are summarized in **Table S8**.

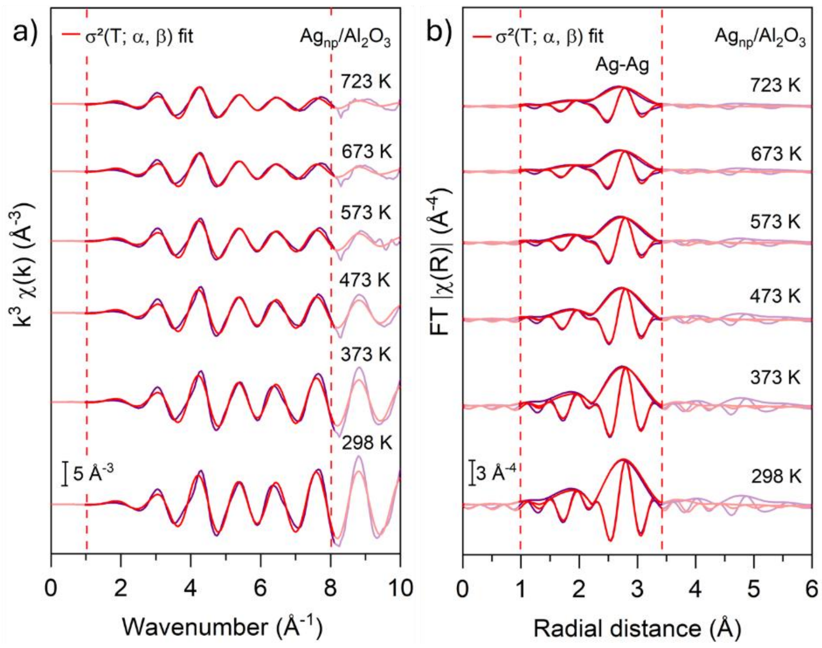

Figure S11. Fit of temperature-resolved Ag K-edge EXAFS spectra for the Ag_{np}/Al_2O_3 catalyst using the linear parameterization σ^2(T; α, β) from Equation 3 (main text). a) k^3-weighted χ(k) EXAFS spectra in the reciprocal k-space and b) their corresponding Fourier transforms FT-|χ(R)| (uncorrected for phase shift). Red solid lines represent the fitted spectra. The k-range defined for the Fourier transform and the R-range used for fitting are indicated by red dashed lines. Structural parameters derived from the fitting are summarized in **Table S9.**

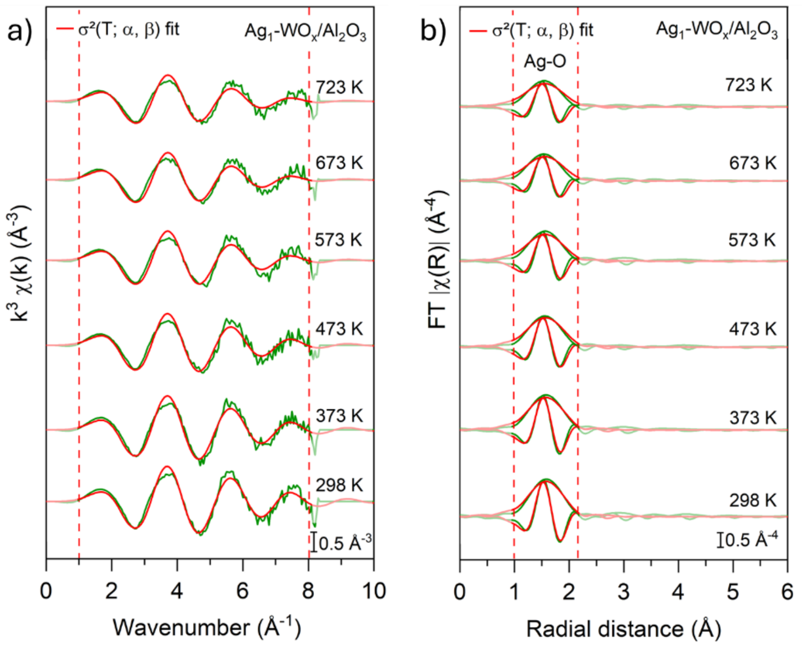

Figure S12. Fit of temperature-resolved Ag K-edge EXAFS spectra for the Ag_1-WO_x/Al_2O_3 catalyst using the linear parameterization σ^2 (T; α, β) from Equation 3 (main text). a) k^3-weighted χ(k) EXAFS spectra in the reciprocal k-space and b) their corresponding Fourier transforms FT-|χ(R)| (uncorrected for phase shift). Red solid lines represent the fitted spectra. The k-range defined for the Fourier transform and the R-range used for fitting are indicated by red dashed lines. Structural parameters derived from the fitting are summarized in **Table S10**.

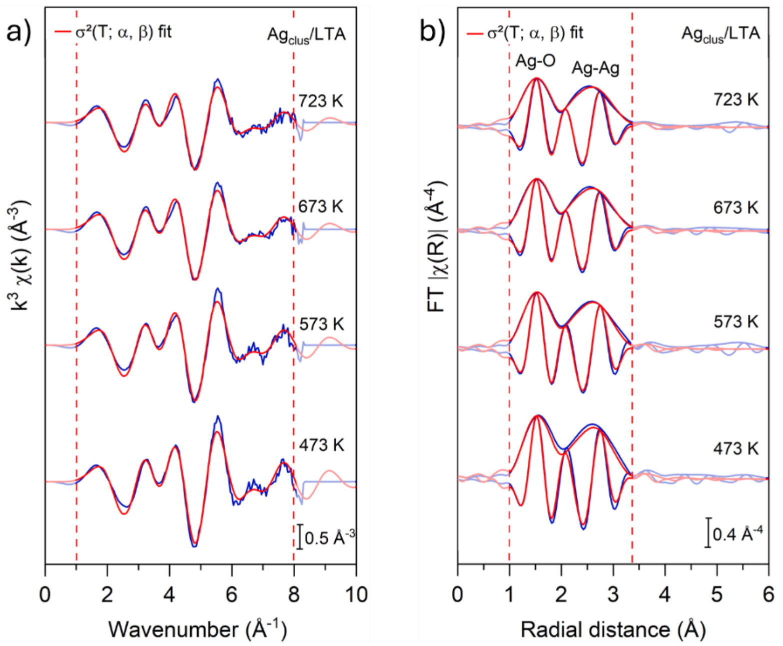

Figure S13. Fit of temperature-resolved Ag K-edge EXAFS spectra for the Ag_{clus}/LTA catalyst using the linear parameterization σ^2 (T; α, β) from Equation 3 (main text). a) k^3-weighted χ(k) EXAFS spectra in the reciprocal k-space and b) their Fourier transforms FT-|χ(R)| (uncorrected for phase shift). Red solid lines represent the fitted spectra. The k-range defined for the Fourier transform and the R-range used for fitting are indicated by red dashed lines. Structural parameters derived from the fitting are summarized in **Table S11**.

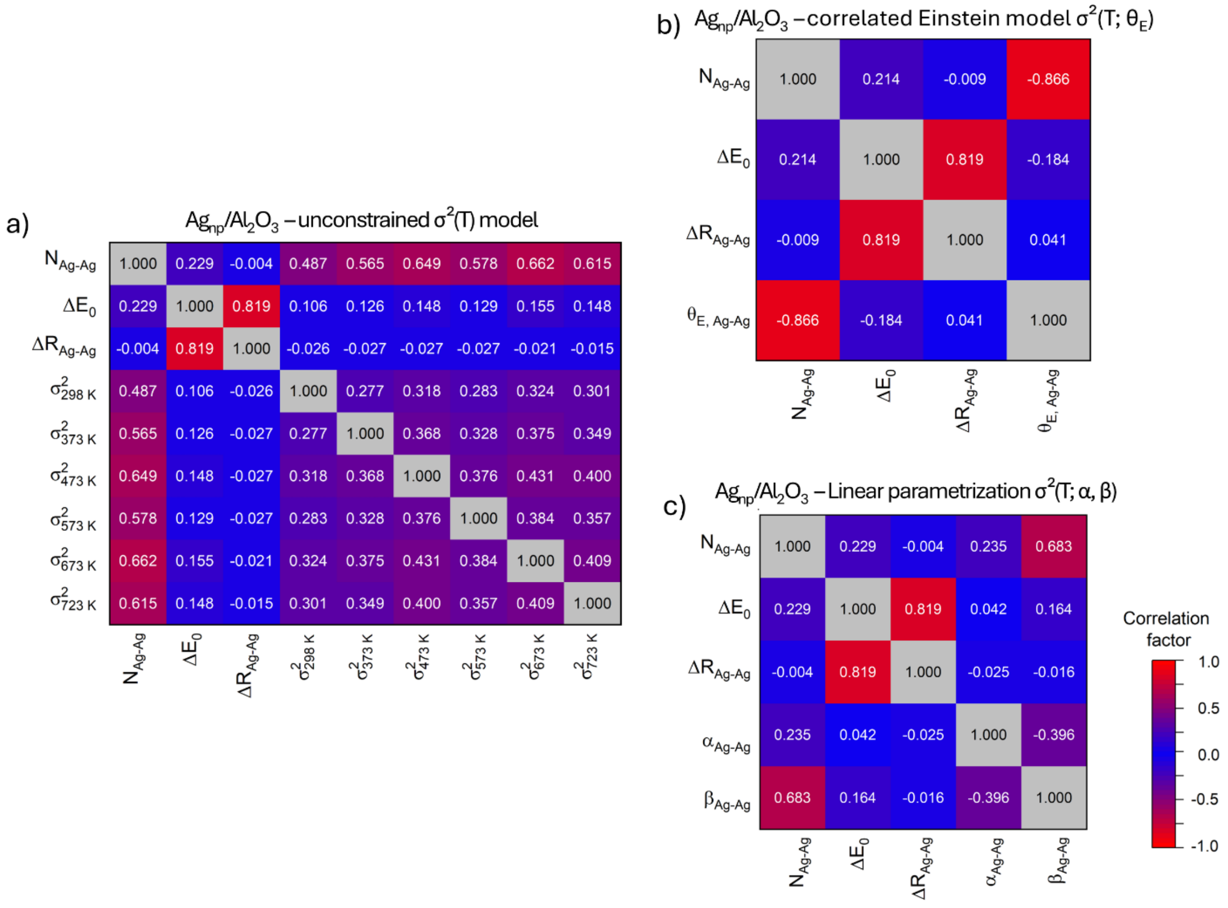

Figure S14. Correlation matrices of the fitting parameters obtained from the temperature-resolved EXAFS analysis of the Ag_{np}/Al_2O_3 catalyst using three fitting approaches: a) unconstrained model ($\sigma^2(T)$, Equation 1 (main text)), b) correlated Einstein model (σ^2 (T; θ_E), Equation 2 (main text)), and c) linear parametrization σ^2 (T; α, β), Equation 3 (main text)). Further details on the structural parameters derived from the fitting, see **Tables S3, S6, S9**.

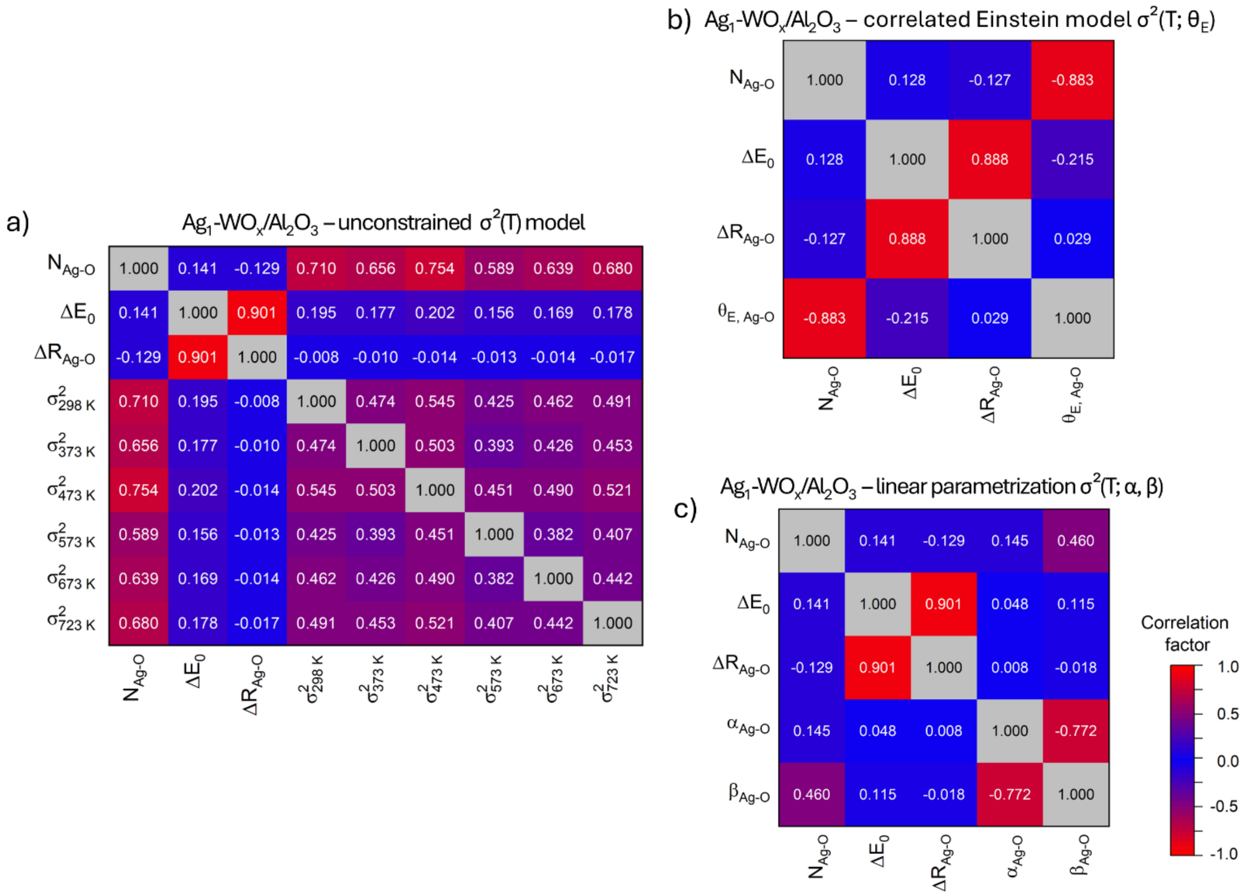

Figure S15. Correlation matrices of the fitting parameters obtained from the temperature-resolved EXAFS analysis of the Ag_1-WO_x/Al_2O_3 catalyst using three fitting approaches: a) unconstrained model ($\sigma^2(T)$, Equation 1 (main text)), b) correlated Einstein model (σ^2 (T; θ_E), Equation 2 (main text)), and c) linear parametrization σ^2 (T; α, β), Equation 3 (main text)). Further details on the structural parameters derived from the fitting, see **Tables S4, S7, S10**.

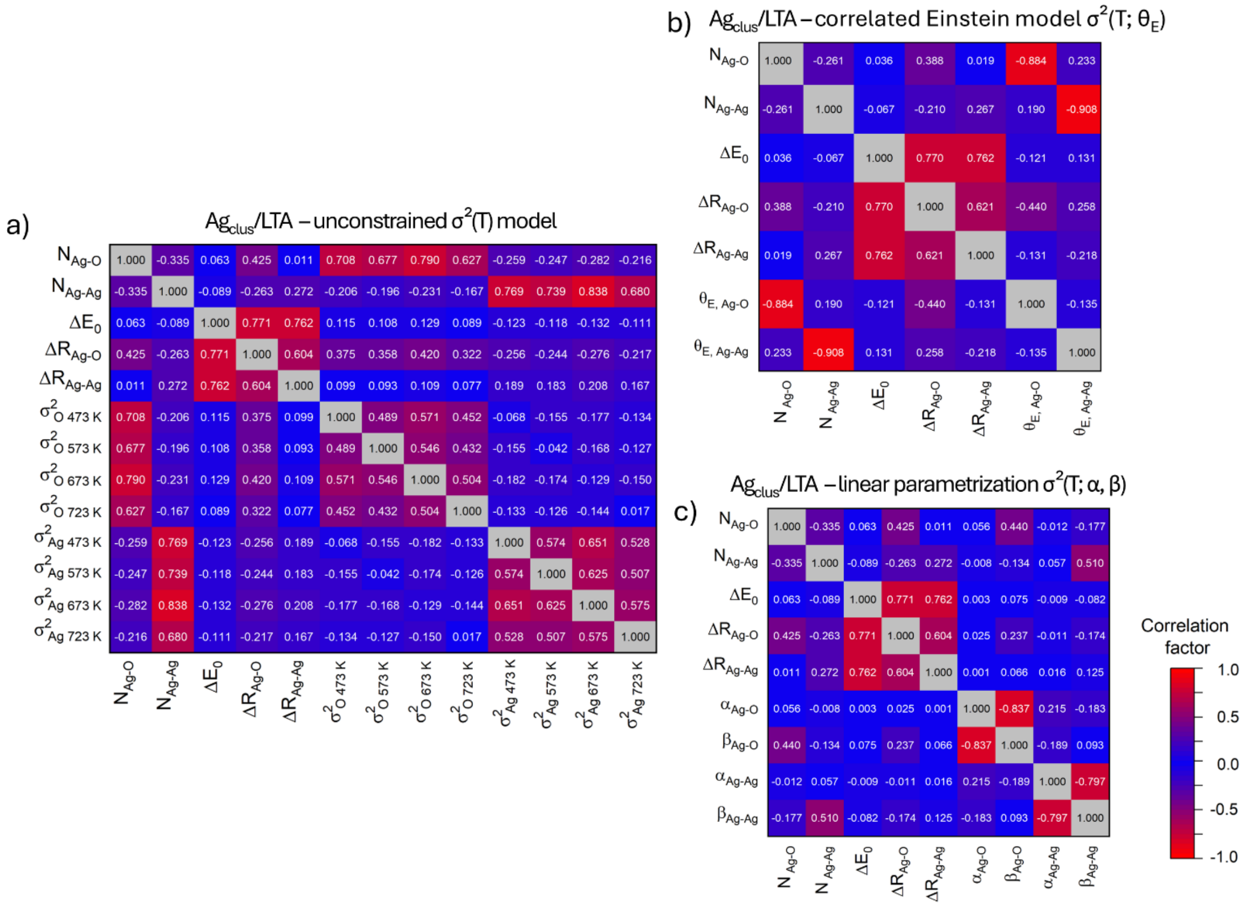

Figure S16. Correlation matrices of the fitting parameters obtained from the temperature-resolved EXAFS analysis of the Ag_{clus}/LTA catalyst using three fitting approaches. a) unconstrained model ($\sigma^2(T)$, Equation 1 (main text)), b) correlated Einstein model (σ^2 (T; θ_E), Equation 2 (main text)), and c) linear parametrization σ^2 (T; α, β), Equation 3 (main text)). Further details on the structural parameters derived from the fitting, see **Tables S5, S8, S11**.

Supporting Tables

Table S1. Summary of the EXAFS fitting parameters for the as-synthesized model catalysts and after the thermal treatment using Equation 1 (main text). N indicates the coordination number, σ^2 is the Debye–Waller factor, ΔR corresponds to the bond length shift, ΔE_0 accounts for the energy shift, and the R-factor quantifies the discrepancy between experimental and fitted data. The fit was performed with simulated single scattering paths for Ag–O (R_{eff} = 2.095 Å) and Ag–Ag (R_{eff} = 2.889 Å) as appropriate for each catalyst. Number of fitting parameters (n_{par}) = $N_{Ag–O}$, $N_{Ag–Ag}$, $\sigma^2_{Ag–O}$, $\sigma^2_{Ag–Ag}$, $\Delta R_{Ag–O}$, $\Delta R_{Ag–Ag}$, ΔE_0. Number of independent points (n_{ind}) = $n_{spectra}$*(2Δk*ΔR)/π. The R-ranges used for the fitting are specified below the table. For graphical representations of the experimental and fitted EXAFS spectra, refer to **Figure S3**. The amplitude reduction factor S_0^2 = 0.94 was determined using a silver foil standard.

Material	State	Path	N	σ^2 (Å^2)	ΔR (Å)	ΔE_0 (eV)	n_{par}/n_{ind}	R-factor
Ag_{np}/Al_2O_3 [a]	as-synthesized	Ag–Ag	12 ± 1	0.013 ± 0.002	-0.021 ± 0.008	1.9 ± 0.4	7/21	0.019
	after treatment		12 ± 1	0.013 ± 0.002	-0.023 ± 0.007			
Ag_1-WO_x/Al_2O_3 [b]	as-synthesized	Ag–O	3.2 ± 0.7	0.023 ± 0.007	0.15 ± 0.03	-0.1 ± 1.3	7/9.5	0.013
	after treatment		3.1 ± 0.7	0.022 ± 0.006	0.15 ± 0.03			
Ag_{clus}/LTA [c]	as-synthesized	Ag–O	3.0 ± 0.8	0.016 ± 0.007	0.19 ± 0.02	-1.1 ± 0.8	13/22	0.042
		Ag–Ag	3.1 ± 1.3	0.017 ± 0.009	-0.09 ± 0.03			
	after treatment	Ag–O	2.9 ± 0.7	0.019 ± 0.006	0.15 ± 0.02			
		Ag–Ag	4.7 ± 1.7	0.027 ± 0.009	-0.09 ± 0.03			

[a] R-range = 1.0-3.4 Å. [b] R-range = 1.0-2.1 Å. [c] R-range = 1.0-3.5 Å.

Table S2. Model catalytic reactions performed over Ag_{np}/Al_2O_3 and Ag_1-WO_x/Al_2O_3 catalysts, demonstrating their functional relevance and catalytic performance.

Catalyst	Reaction	Catalytic performance
Ag_{np}/Al_2O_3	Ethylene epoxidation<br>$C_2H_4 + ½O_2 \longrightarrow CH_2CH_2O$	• $X_{C_2H_4}$ = 3.9 %<br>• $S_{CH_2CH_2O}$= 57.4 % (C-base)<br>• $r_{CH_2CH_2O}$ = 2.3 mmol·g_{Cat}^{-1} · h^{-1}
Ag_1-WO_x/Al_2O_3	Carboxylation of phenylacetylene with CO_2<br>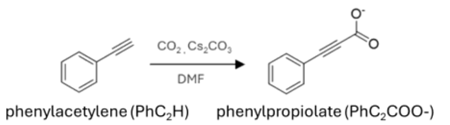	• X_{PhC_2H} = 26 %<br>• $S_{PhCH_2COO^-}$= >95 %<br>• $r_{PhCH_2COO^-}$= 340 mmol·g_{Cat}^{-1}·g_{Ag}^{-1}·h^{-1}

Table S3. Summary of the structural parameters derived from the fitting of the temperature-resolved Ag K-edge EXAFS signals for the Ag_{np}/Al_2O_3 catalyst using the unconstrained $\sigma^2(T)$ model from Equation 1 (main text). N indicates the coordination number, σ^2 is the Debye–Waller factor, ΔR corresponds to the bond length shift, ΔE_0 accounts for the energy shift, and the R-factor quantifies the discrepancy between the experimental and fitted data. The fit was performed using a simulated single scattering path for Ag–Ag (R_{eff} = 2.889 Å) within the ranges 1.0 < k(Å^{-1}) < 8.0 and 1.0 < R(Å) < 3.4. Number of fitting parameters (n_{par}) = $N_{Ag–Ag}$, σ^2_T (T = 298-723 K), $\Delta R_{Ag–Ag}$, ΔE_0. Number of independent points (n_{ind}) = $n_{spectra}$*(2Δk*ΔR)/π. For graphical representations of the experimental and fitted EXAFS spectra, refer to **Figure S5**. The amplitude reduction factor S_0^2 = 0.94 was determined using a silver foil standard.

<table>
<tr><th>T (K)</th><th>Path</th><th>N</th><th>σ² (Å²)</th><th>ΔR (Å)</th><th>ΔE₀ (eV)</th><th>n_par/n_ind</th><th>R-factor</th><th>χ_v²</th></tr>
<tr><td>298</td><td rowspan="6">Ag–Ag</td><td rowspan="6">12 ± 0.6</td><td>0.013 ± 0.001</td><td rowspan="6">-0.021 ± 0.005</td><td rowspan="6">1.7 ± 0.3</td><td rowspan="6">9/62.7</td><td rowspan="6">0.036</td><td rowspan="6">1.01</td></tr>
<tr><td>373</td><td>0.015 ± 0.001</td></tr>
<tr><td>473</td><td>0.019 ± 0.002</td></tr>
<tr><td>573</td><td>0.022 ± 0.002</td></tr>
<tr><td>673</td><td>0.026 ± 0.002</td></tr>
<tr><td>723</td><td>0.028 ± 0.002</td></tr>
</table>

Table S4. Summary of the structural parameters derived from the fitting of the temperature-resolved Ag K-edge EXAFS signals for the Ag_1-WO_x/Al_2O_3 catalyst using the unconstrained $\sigma^2(T)$ model from Equation 1 (main text). N indicates the coordination number, σ^2 is the Debye–Waller factor, ΔR corresponds to the bond length shift, ΔE_0 accounts for the energy shift, and the R-factor quantifies the discrepancy between the experimental and fitted data. The fit was performed using a simulated single scattering path for Ag–O (R_{eff} = 2.095 Å) within the ranges 1.0 < k(Å^{-1}) < 8.0 and 1.0 < R(Å) < 2.1. Number of fitting parameters (n_{par}) = $N_{Ag–O}$, σ^2_T (T = 298-723 K), $\Delta R_{Ag–O}$, ΔE_0. Number of independent points (n_{ind}) = $n_{spectra}$*(2Δk*ΔR)/π. For graphical representations of the experimental and fitted EXAFS spectra, refer to **Figure S6**. The amplitude reduction factor S_0^2 = 0.94 was determined using a silver foil standard.

<table>
<tr><th>T (K)</th><th>Path</th><th>N</th><th>σ² (Å²)</th><th>ΔR (Å)</th><th>ΔE₀ (eV)</th><th>n_par/n_ind</th><th>R-factor</th><th>χ_v²</th></tr>
<tr><td>298</td><td rowspan="6">Ag–O</td><td rowspan="6">3.2 ± 0.2</td><td>0.021 ± 0.003</td><td rowspan="6">0.15 ± 0.01</td><td rowspan="6">-0.8 ± 0.6</td><td rowspan="6">9/28.5</td><td rowspan="6">0.022</td><td rowspan="6">1.34</td></tr>
<tr><td>373</td><td>0.023 ± 0.003</td></tr>
<tr><td>473</td><td>0.026 ± 0.003</td></tr>
<tr><td>573</td><td>0.027 ± 0.003</td></tr>
<tr><td>673</td><td>0.029 ± 0.003</td></tr>
<tr><td>723</td><td>0.030 ± 0.003</td></tr>
</table>

Table S5. Summary of the structural parameters derived from the fitting of the temperature-resolved EXAFS signals for the Ag_{clus}/LTA catalyst using the unconstrained σ^2(T) model from Equation 1 (main text). N indicates the coordination number, σ^2 is the Debye–Waller factor, ΔR corresponds to the bond length shift, ΔE_0 accounts for the energy shift, and the R-factor quantifies the discrepancy between the experimental and fitted data. The fit was performed using simulated single scattering paths for Ag–O (R_{eff} = 2.095 Å) and Ag–Ag (R_{eff} = 2.889 Å) within the ranges 1.0 < k(Å^{-1}) < 8.0 and 1.0 < R(Å) < 3.4. Number of fitting parameters (n_{par})= $N_{Ag–O}$, $N_{Ag–Ag}$, $\sigma^2_{T_{Ag–O,\ Ag–Ag}}$ (T = 473-723 K), $\Delta R_{Ag–O}$, $\Delta R_{Ag–Ag}$, ΔE_0. Number of independent points (n_{ind}) = $n_{spectra}$*(2Δk*ΔR)/π. For graphical representations of the experimental and fitted EXAFS spectra, refer to **Figure S7**. The amplitude reduction factor S_0^2 = 0.94 was determined using a silver foil standard.

T (K)	Path	N	σ^2 (Å^2)	ΔR (Å)	ΔE_0 (eV)	n_{par}/n_{ind}	R-factor	χ_v^2
473	Ag–O	2.1 ± 0.2	0.016 ± 0.003	0.14 ± 0.01	-1.3 ± 0.4	13/41.8	0.026	1.91
573			0.018 ± 0.004					
673			0.019 ± 0.003					
723			0.020 ± 0.004					
473	Ag–Ag	4.9 ± 0.7	0.031 ± 0.005	-0.10 ± 0.01				
573			0.033 ± 0.005					
673			0.035 ± 0.005					
723			0.036 ± 0.006					

Table S6. Summary of the structural parameters derived from the fitting of the temperature-resolved Ag K-edge EXAFS signals for the Ag_{np}/Al_2O_3 catalyst using the correlated Einstein model σ^2 (T; θ_E) from Equation 2 (main text). N indicates the coordination number, θ_E represents the Einstein temperature, σ^2 is the Debye–Waller factor, ΔR corresponds to the bond length shift, ΔE_0 accounts for the energy shift, and the R-factor quantifies the discrepancy between the experimental and fitted data. The fit was performed using a simulated single scattering path for Ag–Ag (R_{eff} = 2.889 Å) within the ranges 1.0 < k(Å^{-1}) < 8.0 and 1.0 < R(Å) < 3.4. Number of fitting parameters (n_{par}) = $N_{Ag–Ag}$, $\theta_{E_{Ag–Ag}}$, $\Delta R_{Ag–Ag}$, ΔE_0. The Debye–Waller factors σ^2_T (T = 298-723 K) were defined using **Equation 2** (main text), with the corresponding θ_E obtained from the global fit and shared across all temperatures. Number of independent points (n_{ind}) = $n_{spectra}$*(2Δk*ΔR)/π. For graphical representations of the experimental and fitted EXAFS spectra, refer to **Figure S8**. The amplitude reduction factor S_0^2 = 0.94 was determined using a silver foil standard.

T (K)	Path	N	θ_E (K)	σ^2 (Å^2)	ΔR (Å)	ΔE_0 (eV)	n_{par}/n_{ind}	R-factor	χ_v^2
298	Ag–Ag	12 ± 0.6	153 ± 4	0.012 ± 0.001	-0.022 ± 0.005	1.7 ± 0.3	4/62.7	0.037	1.09
373				0.015 ± 0.001					
473				0.018 ± 0.001					
573				0.022 ± 0.001					
673				0.026 ± 0.001					
723				0.028 ± 0.002					

Table S7. Summary of the structural parameters derived from the fitting of the temperature-resolved Ag K-edge EXAFS signals for the Ag_1-WO_x/Al_2O_3 catalyst using the correlated Einstein model σ^2 (T; θ_E) from Equation 2 (main text). N indicates the coordination number, θ_E represents the Einstein temperature, σ^2 is the Debye–Waller factor, ΔR corresponds to the bond length shift, ΔE_0 accounts for the energy shift, and the R-factor quantifies the discrepancy between the experimental and fitted data. The fit was performed using a simulated single scattering path for Ag–O (R_{eff} = 2.095 Å) within the ranges 1.0 < k($Å^{-1}$) < 8.0 and 1.0 < R(Å) < 2.1. Number of fitting parameters (n_{par})= $N_{Ag\text{–}O}$, $\theta_{E_{Ag\text{–}O}}$, $\Delta R_{Ag\text{–}O}$, ΔE_0. The Debye–Waller factors $\sigma^2{}_T$ (T = 298-723 K) were defined using **Equation 2** (main text), with the corresponding θ_E obtained from the global fit and shared across all temperatures. Number of independent points (n_{ind}) = $n_{spectra}$*(2Δk*ΔR)/π. For graphical representations of the experimental and fitted EXAFS spectra, refer to **Figure S9**. The amplitude reduction factor $S_0{}^2$ = 0.94 was determined using a silver foil standard.

<table>
<tr><th>T (K)</th><th>Path</th><th>N</th><th>θ_E (K)</th><th>σ^2 ($Å^2$)</th><th>ΔR (Å)</th><th>ΔE_0 (eV)</th><th>n_{par}/n_{ind}</th><th>R-factor</th><th>$\chi_v{}^2$</th></tr>
<tr><td>298</td><td rowspan="6">Ag–O</td><td rowspan="6">2.8 ± 0.2</td><td rowspan="6">290 ± 15</td><td>0.013 ± 0.001</td><td rowspan="6">0.15 ± 0.01</td><td rowspan="6">-0.7 ± 0.7</td><td rowspan="6">4/28.5</td><td rowspan="6">0.038</td><td rowspan="6">4.13</td></tr>
<tr><td>373</td><td>0.016 ± 0.002</td></tr>
<tr><td>473</td><td>0.020 ± 0.002</td></tr>
<tr><td>573</td><td>0.024 ± 0.003</td></tr>
<tr><td>673</td><td>0.028 ± 0.003</td></tr>
<tr><td>723</td><td>0.030 ± 0.003</td></tr>
</table>

Table S8. Summary of the structural parameters derived from the fitting of the temperature-resolved EXAFS signals for the Ag_{clus}/LTA catalyst using the correlated Einstein model σ^2 (T; θ_E) from Equation 2 (main text). N indicates the coordination number, θ_E represents the Einstein temperature, σ^2 is the Debye–Waller factor, ΔR corresponds to the bond length shift, ΔE_0 accounts for the energy shift, and the R-factor quantifies the discrepancy between the experimental and fitted data. The fit was performed using simulated single scattering paths for Ag–O (R_{eff} = 2.095 Å) and Ag–Ag (R_{eff} = 2.889 Å) within the ranges 1.0 < k($Å^{-1}$) < 8.0 and 1.0 < R(Å) < 3.4. Number of fitting parameters (n_{par}) = $N_{Ag\text{–}O}$, $N_{Ag\text{–}Ag}$, $\theta_{E_{Ag\text{–}O}}$, $\theta_{E_{Ag\text{–}Ag}}$, $\Delta R_{Ag\text{–}O}$, $\Delta R_{Ag\text{–}Ag}$, ΔE_0. The Debye–Waller factors $\sigma^2{}_{T_{Ag\text{–}O,\ Ag\text{–}Ag}}$ (T = 473-723 K) were defined using **Equation 2** (main text), with the corresponding θ_E obtained from the global fit and shared across all temperatures. Number of independent points (n_{ind}) = $n_{spectra}$*(2Δk*ΔR)/π. For graphical representations of the experimental and fitted EXAFS spectra, refer to **Figure S10**. The amplitude reduction factor $S_0{}^2$ = 0.94 was determined using a silver foil standard.

<table>
<tr><th>T (K)</th><th>Path</th><th>N</th><th>θ_E (K)</th><th>σ^2 ($Å^2$)</th><th>ΔR (Å)</th><th>ΔE_0 (eV)</th><th>n_{par}/n_{ind}</th><th>R-factor</th><th>$\chi_v{}^2$</th></tr>
<tr><td>473</td><td rowspan="4">Ag–O</td><td rowspan="4">2.1 ± 0.2</td><td rowspan="4">347 ± 25</td><td>0.014 ± 0.002</td><td rowspan="4">0.15 ± 0.01</td><td rowspan="7">-1.2 ± 0.4</td><td rowspan="7">7/41.8</td><td rowspan="7">0.030</td><td rowspan="7">1.75</td></tr>
<tr><td>573</td><td>0.017 ± 0.002</td></tr>
<tr><td>673</td><td>0.020 ± 0.003</td></tr>
<tr><td>723</td><td>0.021 ± 0.003</td></tr>
<tr><td>473</td><td rowspan="3">Ag–Ag</td><td rowspan="3">4.5 ± 0.6</td><td rowspan="3">132 ± 8</td><td>0.025 ± 0.003</td><td rowspan="3">-0.11 ± 0.01</td></tr>
<tr><td>573</td><td>0.030 ± 0.004</td></tr>
<tr><td>673</td><td>0.035 ± 0.004</td></tr>
</table>

723	0.038 ± 0.005

Table S9. Summary of the structural parameters derived from the fitting of the temperature-resolved Ag K-edge EXAFS signals for the Ag_{np}/Al_2O_3 catalyst using the linear parameterization σ^2 (T; α, β) from Equation 3 (main text). N indicates the coordination number, σ^2 is the Debye–Waller factor, α_j and β_j are the linear coefficients obtained from the ordinary least squares (OLS) regression of σ^2 (T), ΔR corresponds to the bond length shift, ΔE_0 accounts for the energy shift, and the R-factor quantifies the discrepancy between the experimental and fitted data. The fit was performed using a simulated single scattering path for Ag–Ag (R_{eff} = 2.889 Å) within the ranges 1.0 < k(Å^{-1}) < 8.0 and 1.0 < R(Å) < 3.4. Number of fitting parameters (n_{par}) = $N_{Ag–Ag}$, $\alpha_{Ag–Ag}$, $\beta_{Ag–Ag}$, $\Delta R_{Ag–Ag}$, ΔE_0. The Debye–Waller factors σ^2_T (T = 298-723 K) were defined using the linear equation $\sigma^2(T) = \alpha_j T + \beta_j$. Number of independent points (n_{ind}) = $N_{spectra}$*(2Δk*ΔR)/π. For graphical representations of the experimental and fitted EXAFS spectra, refer to **Figure S11**. The amplitude reduction factor S_0^2 = 0.94 was determined using a silver foil standard.

T (K)	Path	N	Linear coefficients	σ^2 (Å^2)	ΔR (Å)	ΔE_0 (eV)	n_{par}/n_{ind}	R-factor	χ_v^2
298				0.013 ± 0.001					
373				0.015 ± 0.001					
473	Ag–Ag	12 ± 0.6	$\alpha_{Ag–Ag} = 3.5x10^{-5} \pm 3.4x10^{-6}$ $\beta_{Ag–Ag} = 0.012 \pm 0.001$	0.019 ± 0.001	-0.021 ± 0.005	1.7 ± 0.3	5/62.7	0.037	1.11
573				0.022 ± 0.001					
673				0.026 ± 0.002					
723				0.027 ± 0.002					

Table S10. Summary of the structural parameters derived from the fitting of the temperature-resolved EXAFS signals for the Ag_1-WO_x/Al_2O_3 catalyst using the linear parameterization σ^2(T; α, β) from Equation 3 (main text). N indicates the coordination number, σ^2 is the Debye–Waller factor, α_j and β_j are the linear coefficients obtained from the ordinary least squares (OLS) regression of σ^2 (T), ΔR corresponds to the bond length shift, ΔE_0 accounts for the energy shift, and the R-factor quantifies the discrepancy between the experimental and fitted data. The fit was performed using a simulated single scattering path for Ag–O (R_{eff} = 2.095 Å) within the ranges 1.0 < k(Å^{-1}) < 8.0 and 1.0 < R(Å) < 2.1. Number of fitting parameters (n_{par}) = $N_{Ag–O}$, $\alpha_{Ag–O}$, $\beta_{Ag–O}$, $\Delta R_{Ag–O}$, ΔE_0. The Debye–Waller factors σ^2_T (T = 298-723 K) were defined using the linear equation $\sigma^2(T) = \alpha_j T + \beta_j$. Number of independent points (n_{ind}) = $n_{spectra}$*(2Δk*ΔR)/π. For graphical representations of the experimental and fitted EXAFS spectra, refer to **Figure S12**. The amplitude reduction factor S_0^2 = 0.94 was determined using a silver foil standard.

T (K)	Path	N	Linear coefficients	σ^2 (Å^2)	ΔR (Å)	ΔE_0 (eV)	n_{par}/n_{ind}	R-factor	χ_v^2
298				0.022 ± 0.002					
373				0.023 ± 0.002					
473	Ag–O	3.2 ± 0.2	$\alpha_{Ag–O} = 2.1x10^{-5} \pm 5.2x10^{-6}$ $\beta_{Ag–O} = 0.021 \pm 0.002$	0.025 ± 0.002	0.15 + 0.01	-0.8 ± 0.5	5/28.5	0.022	1.19
573				0.027 ± 0.003					
673				0.029 ± 0.003					
723				0.030 ± 0.003					

Table S11. Summary of the structural parameters derived from the fitting of the temperature-resolved EXAFS signals for the Ag_{clus}/LTA catalyst using the linear parameterization σ^2 (T; α, β) from Equation 3 (main text). N indicates the coordination number, σ^2 is the Debye–Waller factor, α_j and β_j are the linear coefficients obtained from the ordinary least squares (OLS) regression of σ^2 (T), ΔR corresponds to the bond length shift, ΔE_0 accounts for the energy shift, and the R-factor quantifies the discrepancy between the experimental and fitted data. The fit was performed using simulated single scattering path for Ag–O (R_{eff} = 2.095 Å) and Ag–Ag (R_{eff} = 2.889 Å) within the ranges 1.0 < k(Å^{-1}) < 8.0 and 1.0 < R(Å) < 3.4. Number of fitting parameters (n_{par}) = $N_{Ag–O}$, $N_{Ag–Ag}$, $\alpha_{Ag–O}$, $\beta_{Ag–O}$, $\alpha_{Ag–Ag}$, $\beta_{Ag–Ag}$, $\Delta R_{Ag–O}$, $\Delta R_{Ag–Ag}$, ΔE_0. The Debye–Waller factors $\sigma^2_{T_{Ag–O,\ Ag–Ag}}$ (T = 298-723 K) were defined using the linear equation $\sigma^2(T) = \alpha_j T + \beta_j$. Number of independent points (n_{ind}) = $n_{spectra}$*(2Δk*ΔR)/π. For graphical representations of the experimental and fitted EXAFS spectra, refer to **Figure S13**. The amplitude reduction factor S_0^2 = 0.94 was determined using a silver foil standard.

T (K)	Path	N	Linear coefficients	σ^2 (Å^2)	ΔR (Å)	ΔE_0 (eV)	N_{par}/n_{ind}	R-factor	χ_v^2
473	Ag–O	2.1 ± 0.2	$\alpha_{Ag–O}$ = 1.8x10^{-5} ± 1.2x10^{-5}	0.016 ± 0.005	0.14 ± 0.01	-1.3 ± 0.4	9/41.8	0.026	1.79
573			$\beta_{Ag–O}$ = 0.012 ± 0.005	0.017 ± 0.006					
673				0.019 ± 0.007					
723				0.020 ± 0.007					
473	Ag–Ag	4.8 ± 0.7	$\alpha_{Ag–Ag}$ = 2.1x10^{-5} ± 1.6x10^{-5}	0.031 ± 0.007	-0.10 ± 0.01				
573			$\beta_{Ag–Ag}$ = 0.027 ± 0.007	0.033 ± 0.008					
673				0.035 ± 0.009					
723				0.036 ± 0.010					
